# Supplementary material for: Structural and evolutionary insights into the eukaryotic RNase MRP ribonucleoprotein complex
Source: Nat Commun. 2026 Mar 26;17:4451. doi: 10.1038/s41467-026-71007-9 (PMC13184271; doi:10.1038/s41467-026-71007-9)
Supplement: Supplementary file 1 — Supplementary Information [file 41467_2026_71007_MOESM1_ESM.pdf]

Supplementary Information for

Structural and Evolutionary Insights into the Eukaryotic RNase MRP Ribonucleoprotein Complex

Bin Zhou[1,2,8], Xiaozhu Wang[2,8], Futang Wan[3,8], Shaobai Li[2], Xiaoshuang Zhang[2], Yuanyuan Zhang[2], Ming Tan[1,2], Mi Cao[2], Yafeng Shen[2], Rui Gao[4], Yanjie Zhang[1*], Pengfei Lan[5,6*], Jian Wu[2*] and Ming Lei[2,6,7*]

[1]Department of Oncology, Ninth People's Hospital, Shanghai Jiao Tong University School of Medicine; Shanghai 201900, China.

[2]Shanghai Institute of Precision Medicine, Ninth People's Hospital, Shanghai Jiao Tong University School of Medicine; Shanghai 200125, China.

[3]Cancer Institute, The Affiliated Hospital of Qingdao University, Qingdao Cancer Institute, Qingdao University, Qingdao 266071, China.

[4]Frontier Science Center for Stem Cell Research, Tongji University, Shanghai 200092, China.

[5]Institute of Aging and Tissue Regeneration, Renji Hospital, Shanghai Jiao Tong University School of Medicine; Shanghai 201900, China.

[6]State Key Laboratory of Systems Medicine for Cancer, Shanghai Jiao Tong University School of Medicine, Shanghai 200125, China.

[7]Shanghai Academy of Natural Sciences (SANS), Shanghai Jiao Tong University, Shanghai 200125, China

[8]These authors contributed equally: Bin Zhou, Xiaozhu Wang and Futang Wan

[*]Corresponding author. E-mail: leim@shsmu.edu.cn (M.L.), wujian@shsmu.edu.cn (J.W.), pengfeilan@shsmu.edu.cn (P.L.), and zhangyanjie@shsmu.edu.cn (Y.Z.).

The PDF file includes:

- Supplementary Figures 1 - 24
- Supplementary Table 1

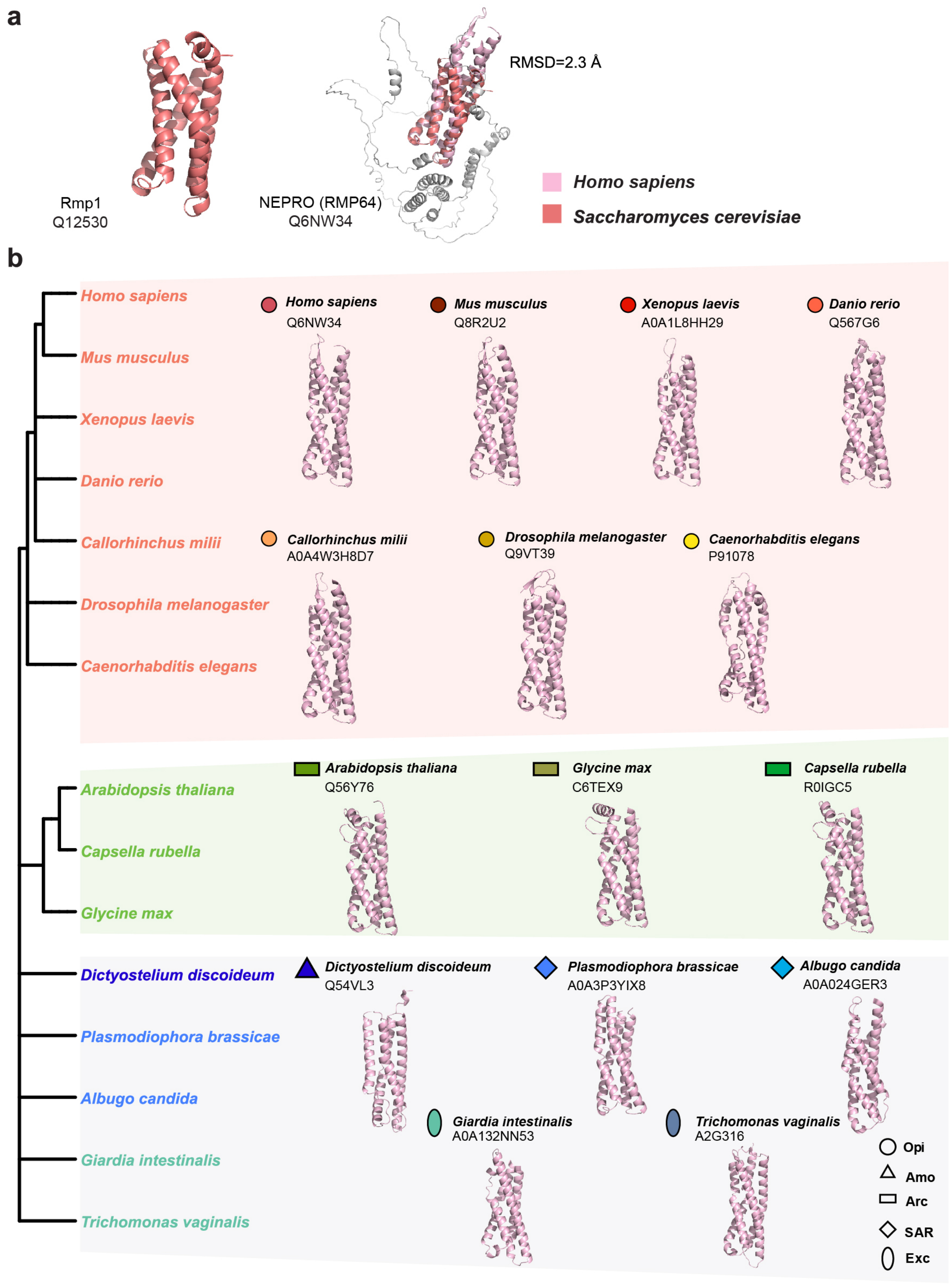

Supplementary Fig. 1 Evolutionary conservation analysis of the NEPRO (RMP64) subunit of RNase MRP. a, Structural conservation of NEPRO (RMP64). Superimposed structures of NEPRO (RMP64) from *Saccharomyces cerevisiae* (Rmp1,

UniProt: Q12530) and *Homo sapiens* (UniProt: Q6NW34) with a root-mean-square deviation (RMSD) of 2.3 Å. **b**, Phylogenetic distribution of NEPRO (RMP64) homologs. The phylogenetic tree (left) illustrates the evolutionary relationships among the representative species. The representative NEPRO (RMP64) structures predicted by AlphaFold-3 are shown. UniProt accession numbers are provided for each species. Major eukaryotic supergroups are abbreviated as follows: Opi: Opisthokonta, Amo: Amoebozoa, Arc: Archaeplastida, SAR: S: Stramenopiles A: Alveolata R: Rhizaria, and Exc: Excavata.

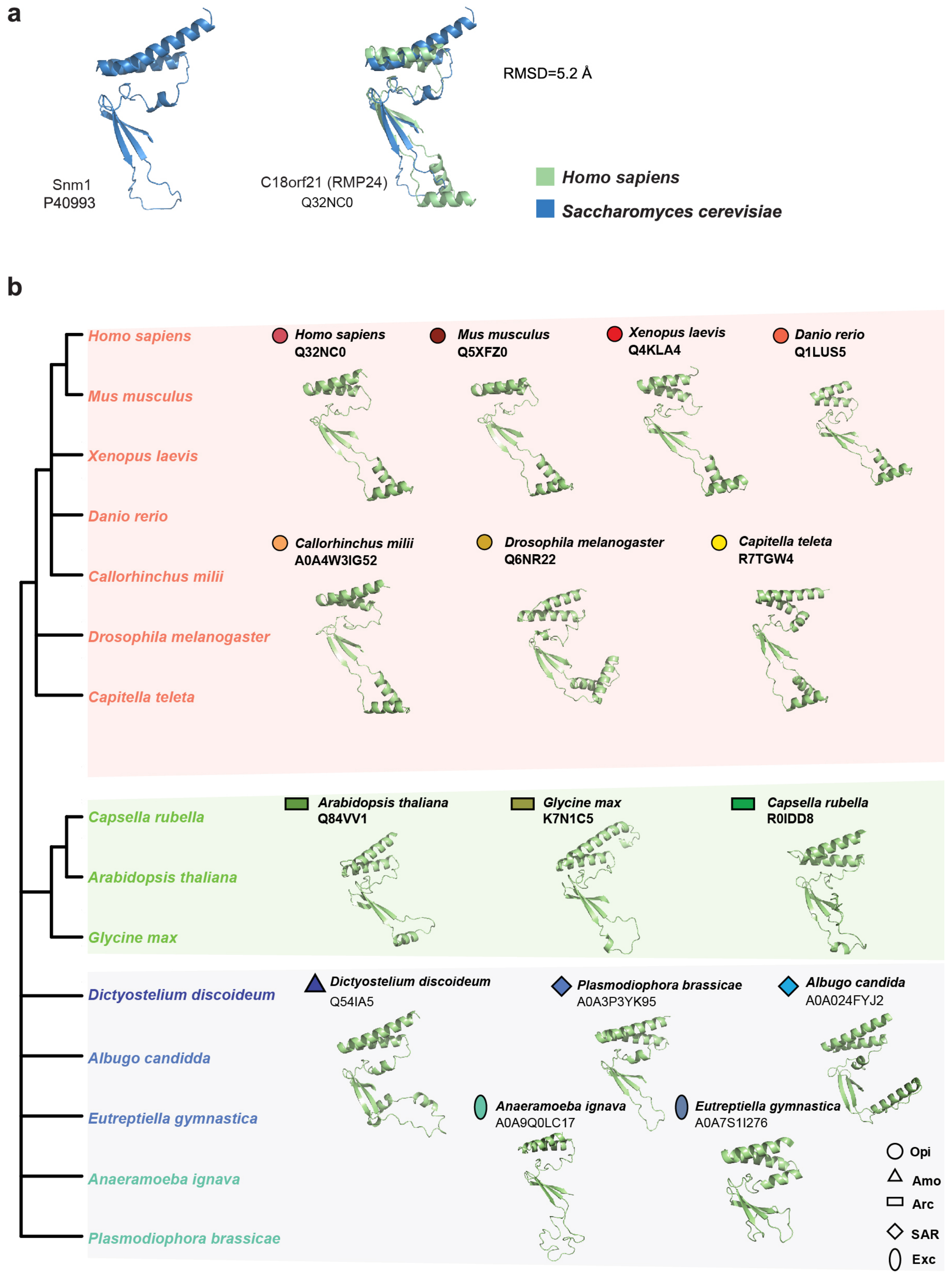

Supplementary Fig. 2 Evolutionary conservation analysis of the C18orf21 (RMP24) subunit of RNase MRP. a, Structural conservation of C18orf21 (RMP24). Superimposed structures of C18orf21 (RMP24) from *Saccharomyces cerevisiae* (Snm1,

UniProt: P40993) and *Homo sapiens* (UniProt: Q32NC0), with an RMSD of 5.2 Å. **b**, Phylogenetic distribution of Snm1 homologs. The phylogenetic tree (left) illustrates the evolutionary relationship among the representative species. The representative C18orf21 (RMP24) structures predicted by AlphaFold-3 are shown. UniProt accession numbers are provided for each species. Major eukaryotic supergroups are abbreviated as follows: Opi: Opisthokonta, Amo: Amoebozoa, Arc: Archaeplastida, SAR: S: Stramenopiles A: Alveolata R: Rhizaria, and Exc: Excavata.

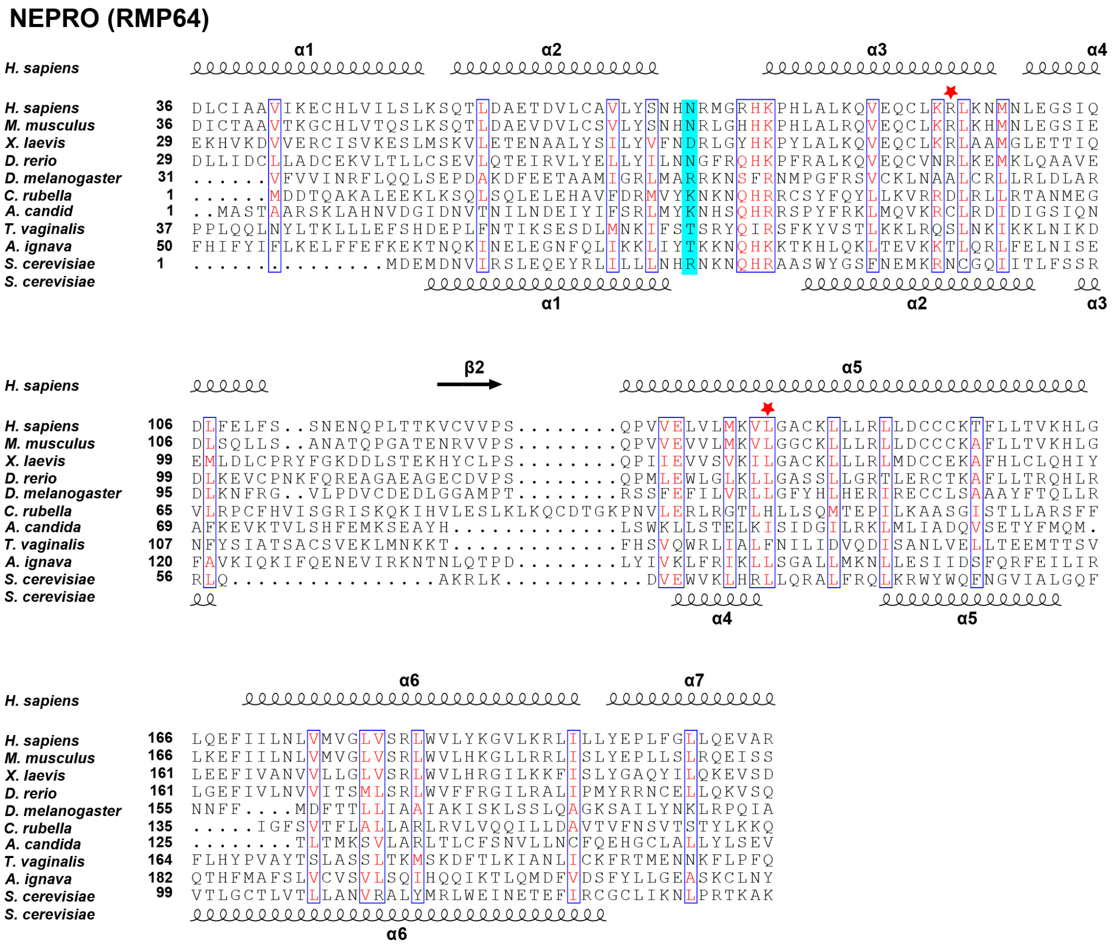

Supplementary Fig. 3 Multiple sequence alignment of the RNase MRP components NEPRO (RMP64). Sequences of NEPRO (RMP64) from representative eukaryotes were aligned. Conserved residues are colored in red. Residues that stabilize the +4 anchor are highlighted in cyan; disease-associated residues are denoted with red asteroids.

C18orf21 (RMP24)

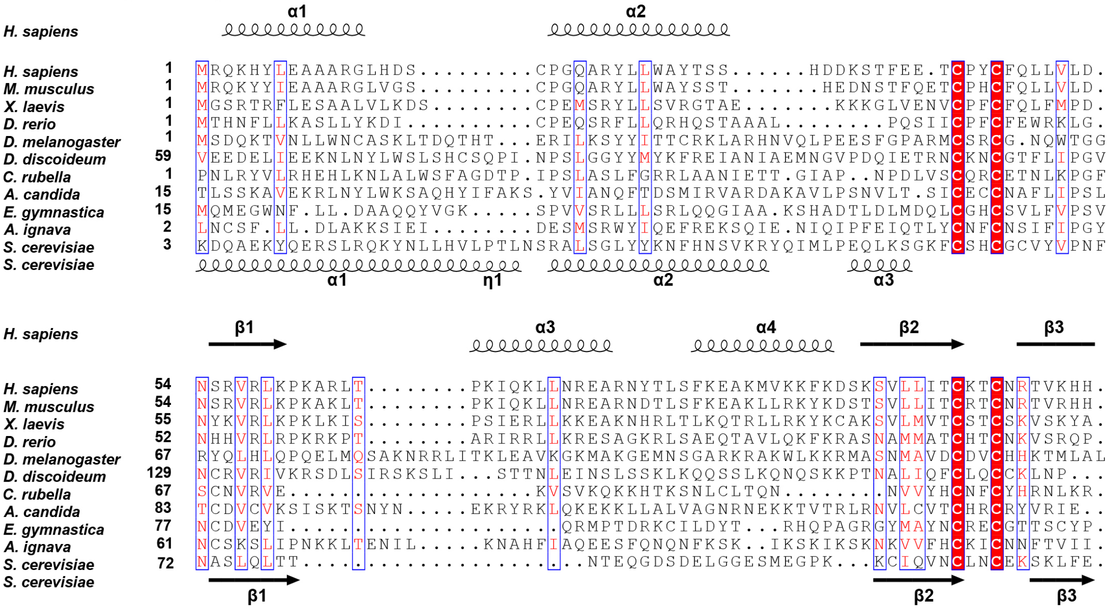

Supplementary Fig. 4 Multiple sequence alignment of the C18orf21 (RMP24) subunit of RNase MRP. Sequences of C18orf21 (RMP24) from representative eukaryotes were aligned. Conserved residues are colored in red. Four zinc-coordinating cysteine residues are highlighted in red.

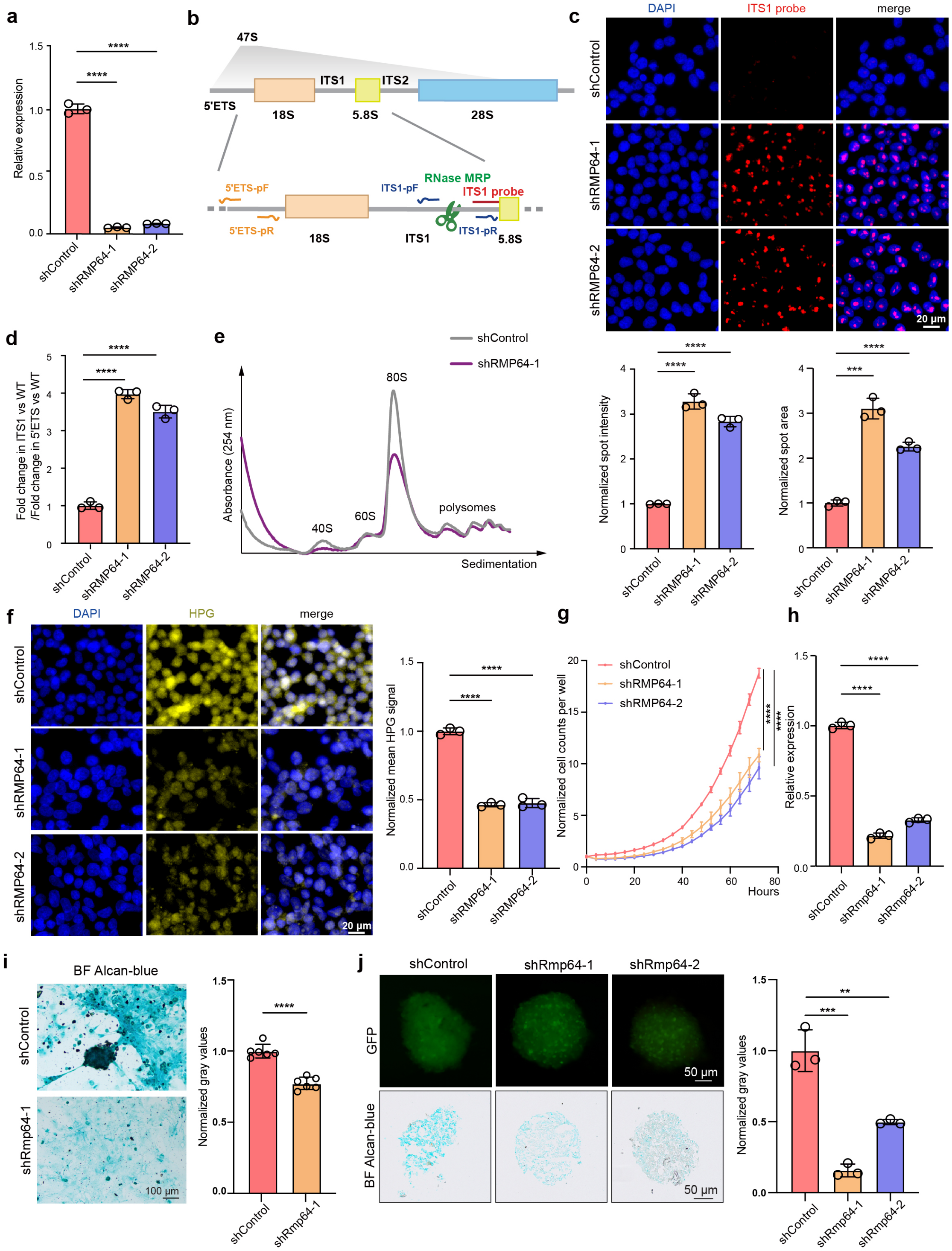

Supplementary Fig. 5 RMP64 is essential for pre-rRNA processing, ribosome assembly, and chondrogenic differentiation. a, Knockdown efficiency of RMP64 shRNA. **b,** Schematic representation of human 47S pre-rRNA (upper panel) and the

enlarged view of the 5' ETS-5.8S region (lower panel). Primer locations for detecting 5' ETS and ITS1 are denoted. The ITS1 FISH probe is indicated. **c,** FISH analysis of pre-rRNA accumulation in HEK293T cells transfected with shControl, shRMP64-1, or shRMP64-2. Quantitative analyses of spot intensity and spot area are shown on the bottom. Scale bar: 20 μm. At least 100 cells were analyzed. **d,** RMP64 knockdown impairs pre-rRNA processing in HEK293T cells. qRT-PCR analysis showing increased pre-cleaved ITS1/5' ETS ratio in RMP64-knockdown cells. **e,** Sucrose gradient profiling shows that RMP64-knockdown cells exhibit reduced levels of 40S and 80S ribosomal subunits compared with control cells. **f,** Immunofluorescence analysis of protein synthesis in HEK293T cells transfected with shControl, shRMP64-1, or shRMP64-2, with the quantification shown on the right. Scale bar, 20 μm. At least 100 cells were analyzed. **g**, Proliferation analysis of control and RMP64 knockdown cells was performed using the Incucyte software, images were acquired every 4 hours, and analyzed by the Cell-by-Cell adherent analysis. Data were shown as mean ± SD from at least three replicates and the final timepoint of the growth curve was calculated using One-way ANOVA. **h,** Knockdown efficiency of Rmp64 shRNA in mouse BMSCs. **i-j,** RMP64-knockdown impairs chondrogenic differentiation. Representative images of alcian blue staining of 2D (**i**) and 3D cultures (**j**) from mouse bone marrow stromal cells showing reduced sulfated glycosaminoglycan deposition in RMP64-knockdown cells. For all experiments, data were shown as mean ± SD from at least three independent replicates. The gray values were analyzed by image J. Significance was determined with a two-tailed Student's t-test. ***$p < 0.001$, ****$p < 0.0001$.

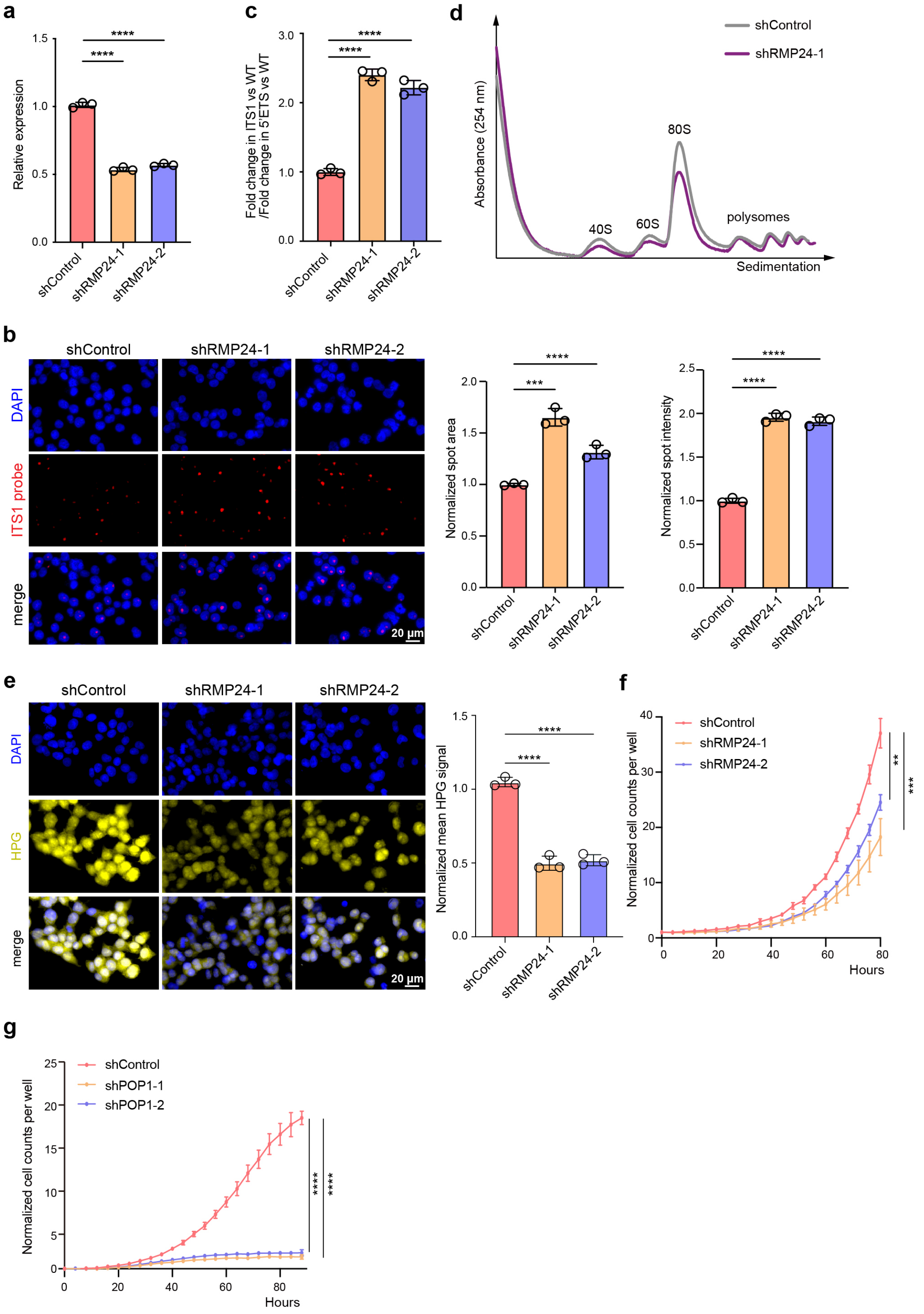

Supplementary Fig. 6 RMP24 is essential for ribosome biogenesis and protein homeostasis. a, Knockdown efficiency of RMP24 shRNA in HEK293T cells. **b**, FISH analysis of pre-rRNA accumulation in shControl, shRMP24-1, and shRMP24-2

HEK293T cells, with the spot intensity and spot area quantification shown on the right. Scale bar, 20 μm. At least 100 cells were analyzed. **c**, Quantitative RT-PCR analysis revealed elevated pre-cleaved ITS1 levels relative to the 5'-external transcribed spacer (5' ETS) in RMP24 knockdown HEK293T cells. **d**, Sucrose gradient profiling shows that RMP24 - knockdown cells exhibit reduced levels of 40S and 80S ribosomal subunits compared with control cells. **e**, IF analysis of protein synthesis in shControl, shRMP24-1, and shRMP24-2 HEK293T cells, with the quantification shown on the right. Scale bar, 20 μm. At least 100 cells were analyzed. **f-g**, Proliferation analysis of control and RMP24 knockdown cells (**f**) or POP1 knockdown cells (**g**) was performed using the Incucyte software, images were acquired every 4 hours, and analyzed by the Cell-by-Cell adherent analysis. For all experiments, data were shown as mean ± SD from at least three independent replicates. Significance was determined with a two-tailed Student's t-test. And the statistical significance at the final timepoint of the growth curves (f, g) was calculated using One-way ANOVA. $^{**}p < 0.01$, $^{***}p < 0.001$, $^{****}p < 0.0001$.

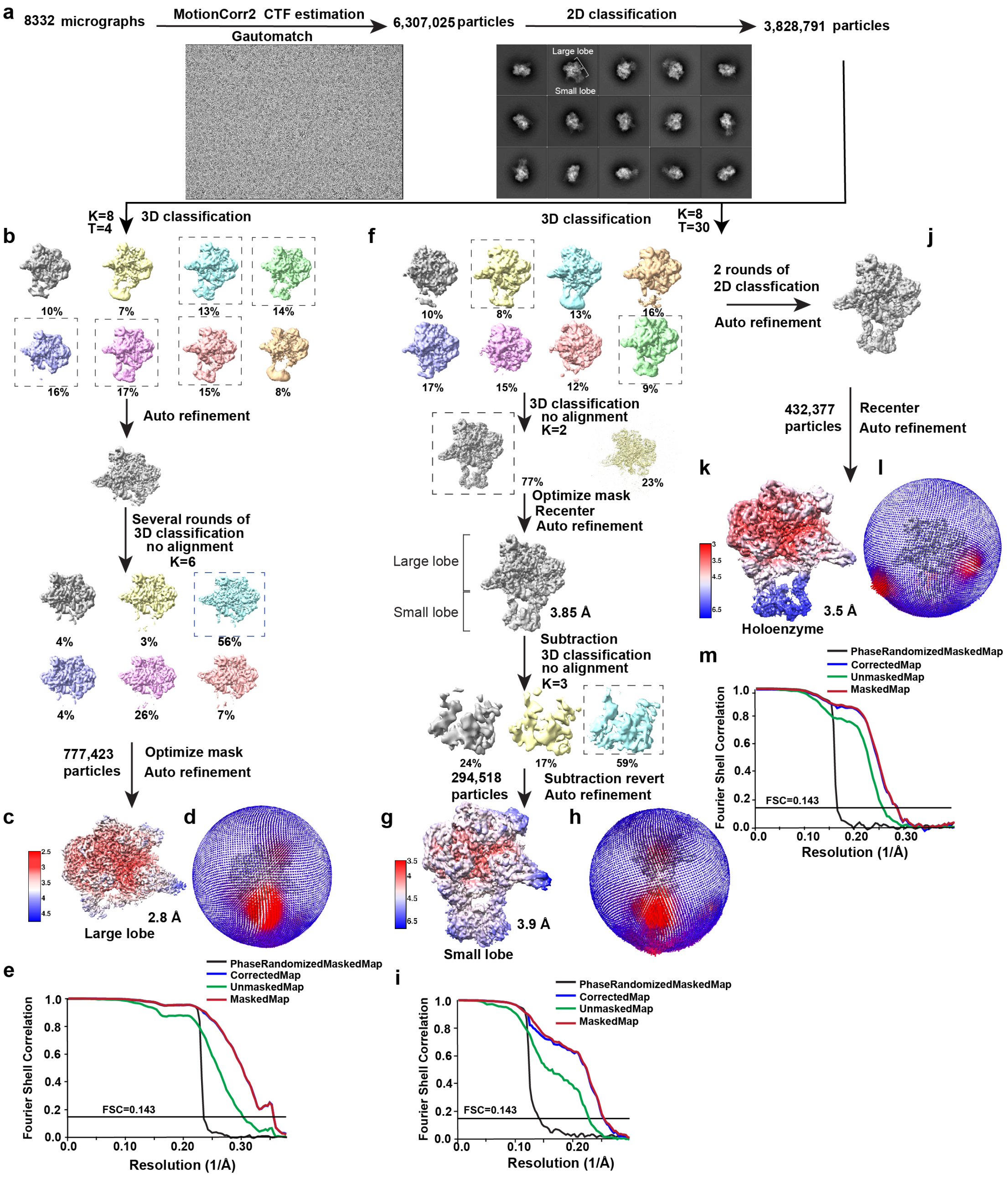

Supplementary Fig. 7 Cryo-EM analysis of human RNase MRP complex. a, Left: A representative raw cryo-EM image of the RNase MRP holoenzyme. Right: 2D class averages of the RNase MRP holoenzyme. **b**, Brief image processing flowchart for the RNase MRP large lobe. **c**, Local resolution map for the RNase MRP large lobe. **d**, Angular distributions of particles for the final reconstruction of the RNase MRP large lobe. **e**, Fourier Shell Correlation curve of the reconstructed map of the RNase MRP

large lobe. **f**, Brief image processing flowchart for the RNase MRP small lobe. **g**, Local resolution map for the RNase MRP small lobe. **h**, Angular distributions of particles for the final reconstruction of the RNase MRP small lobe. **i**, Fourier shell correlation curve of the reconstructed map of the RNase MRP small lobe. **j**, 3D refinement of human RNase MRP holoenzyme complex using C1 symmetry. **k**, Local resolution map for the RNase MRP holoenzyme complex. **l**, Angular distributions of particles for the final reconstruction of the RNase MRP holoenzyme complex. **m**, Fourier shell correlation curve of the reconstructed map of the RNase MRP holoenzyme complex.

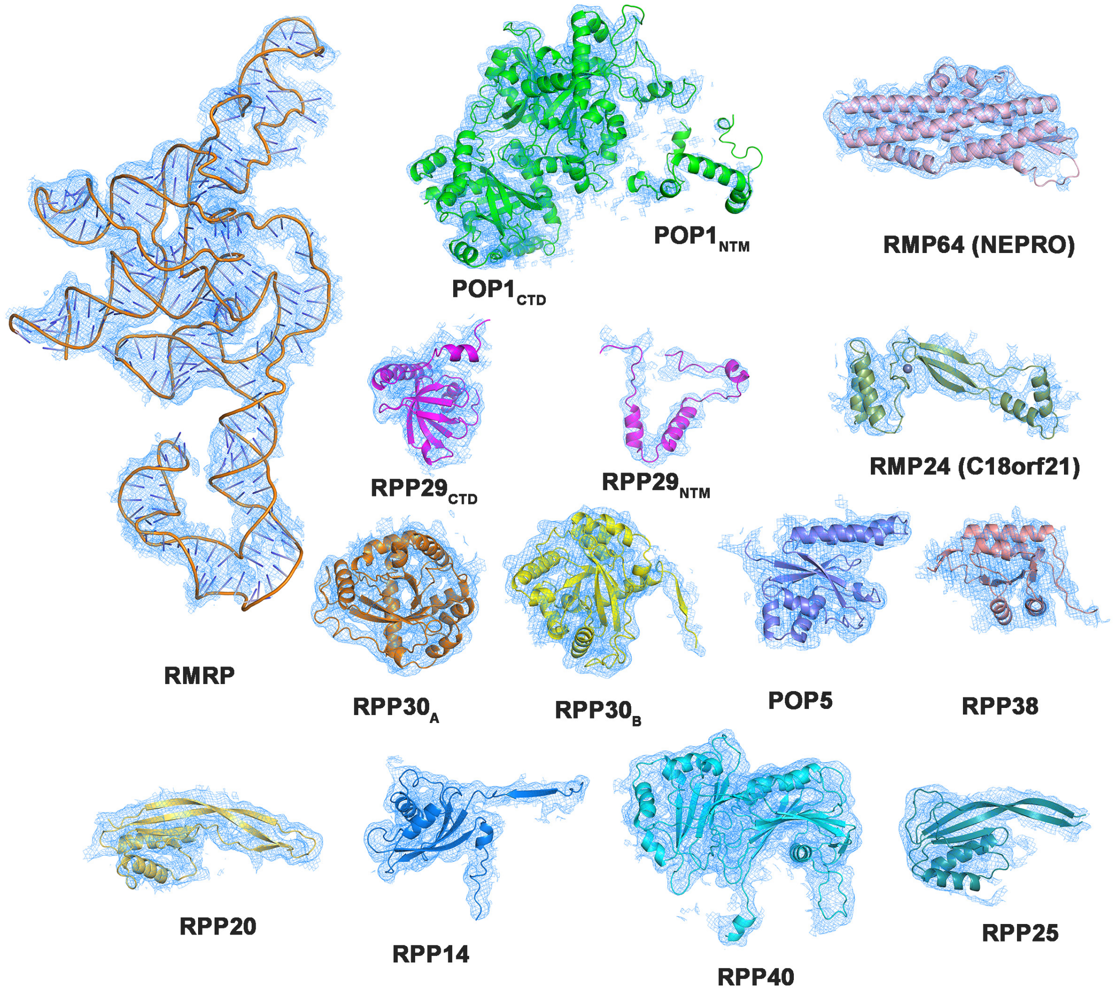

Supplementary Fig. 8 Cryo-EM density maps of individual protein and RNA subunits in the human RNase MRP holoenzyme complex. Cartoons of each structure are fitted into the EM density map and colored as in Fig. 2a.

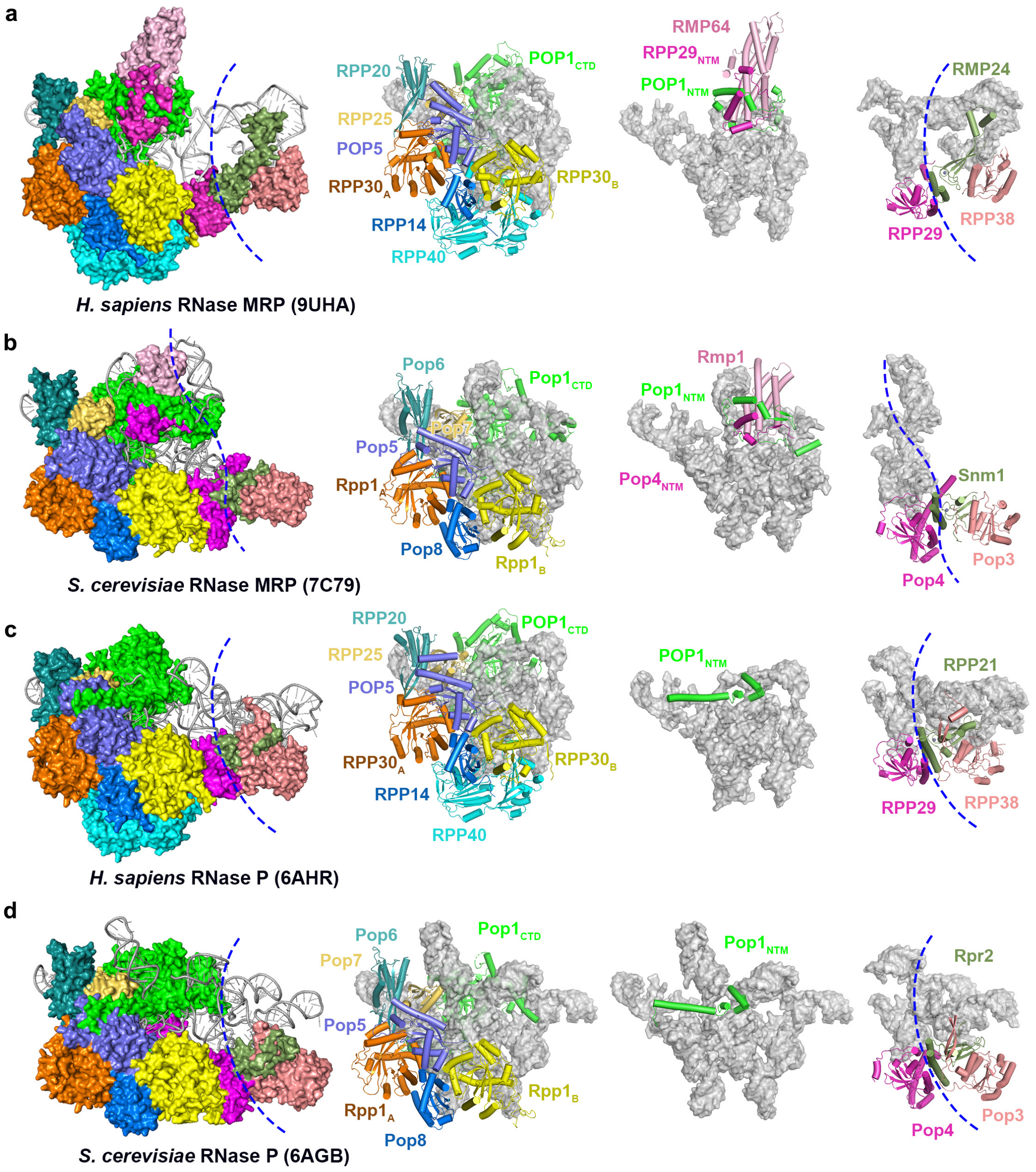

Supplementary Fig. 9 A conserved architectural framework of eukaryotic RNase P/MRP complexes. a, Human RNase MRP structural organization (PDB: 9UHA). First panel: The hook-shaped protein scaffold enwraps the RMRP RNA, with subunits colored as in Fig. 2. Second panel: Large lobe assembly comprising POP1$_{CTD}$, the RPP20-RPP25 heterodimer, and the POP5-(RPP30)$_2$-RPP14-RPP40 heteropentamer enwrapping RMRP. Third panel: POP1$_{NTM}$ and RPP29$_{NTM}$ undergo a marked refolding,

and together with RMP64 form the RMP64-POP1$_{NTM}$-RPP29$_{NTM}$ module. Fourth panel: Small lobe stabilization by the RMP24-RPP29-RPP38 module. **b-d**, Organizations of yeast RNase MRP (PDB: 7C79) (**b**), human RNase P (PDB: 6AHR) (**c**), and yeast RNase P (PDB: 6AGB) (**d**). Each panel corresponds to its counterpart in (**a**), respectively.

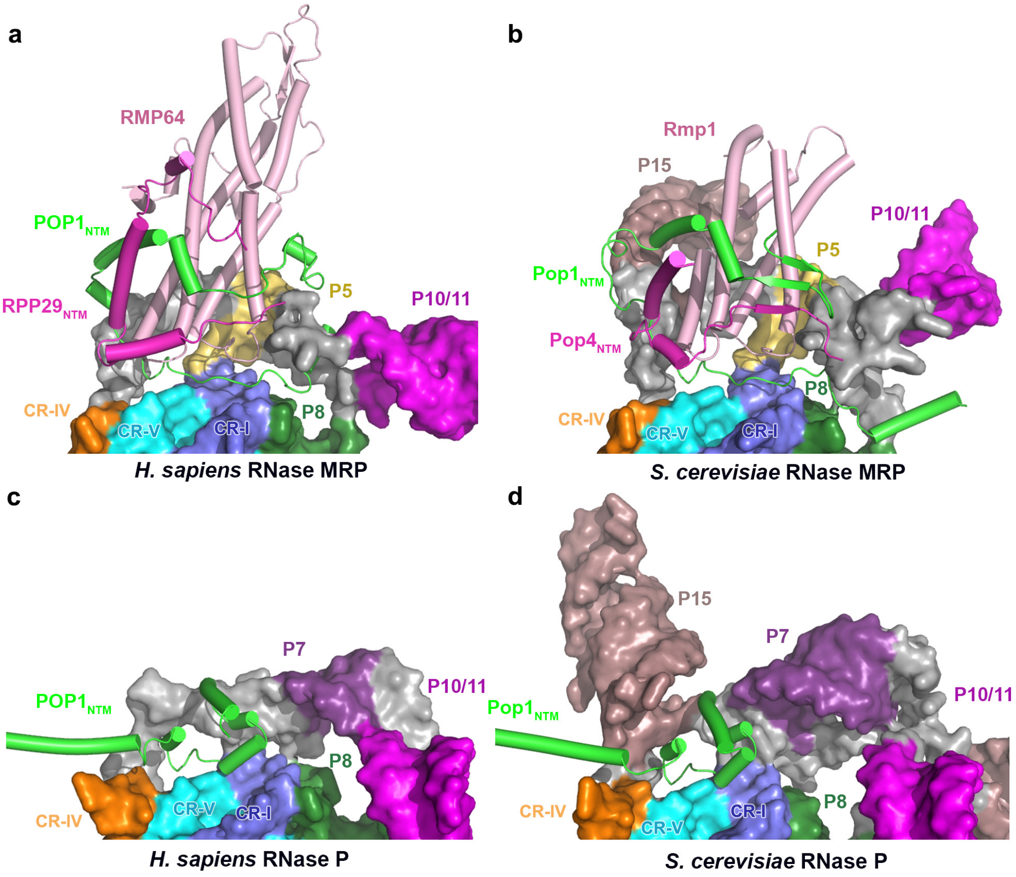

Supplementary Fig. 10 Structural conservation and divergence of the C-domain architecture in RNase MRP RNAs. a-b, Surface representation of the P4-P5 cradle-shaped surface, with the RMP64-POP1$_{NTM}$-RPP29$_{NTM}$ ternary module docked on the RNA platform in human (**a**) and yeast (**b**) RNase MRP. The protein components and RNA elements are shown in cartoon and surface representations respectively and colored as in Fig. 2. **c-d**, The small cavity between CR-IV and stem P7 in human (**c**) and yeast (**d**) RNase P RNAs. The refolded POP1$_{NTM}$ fits in the cavity. POP1$_{NTM}$ and the RNA elements are shown in cartoon and surface representations respectively and colored as in Fig. 2.

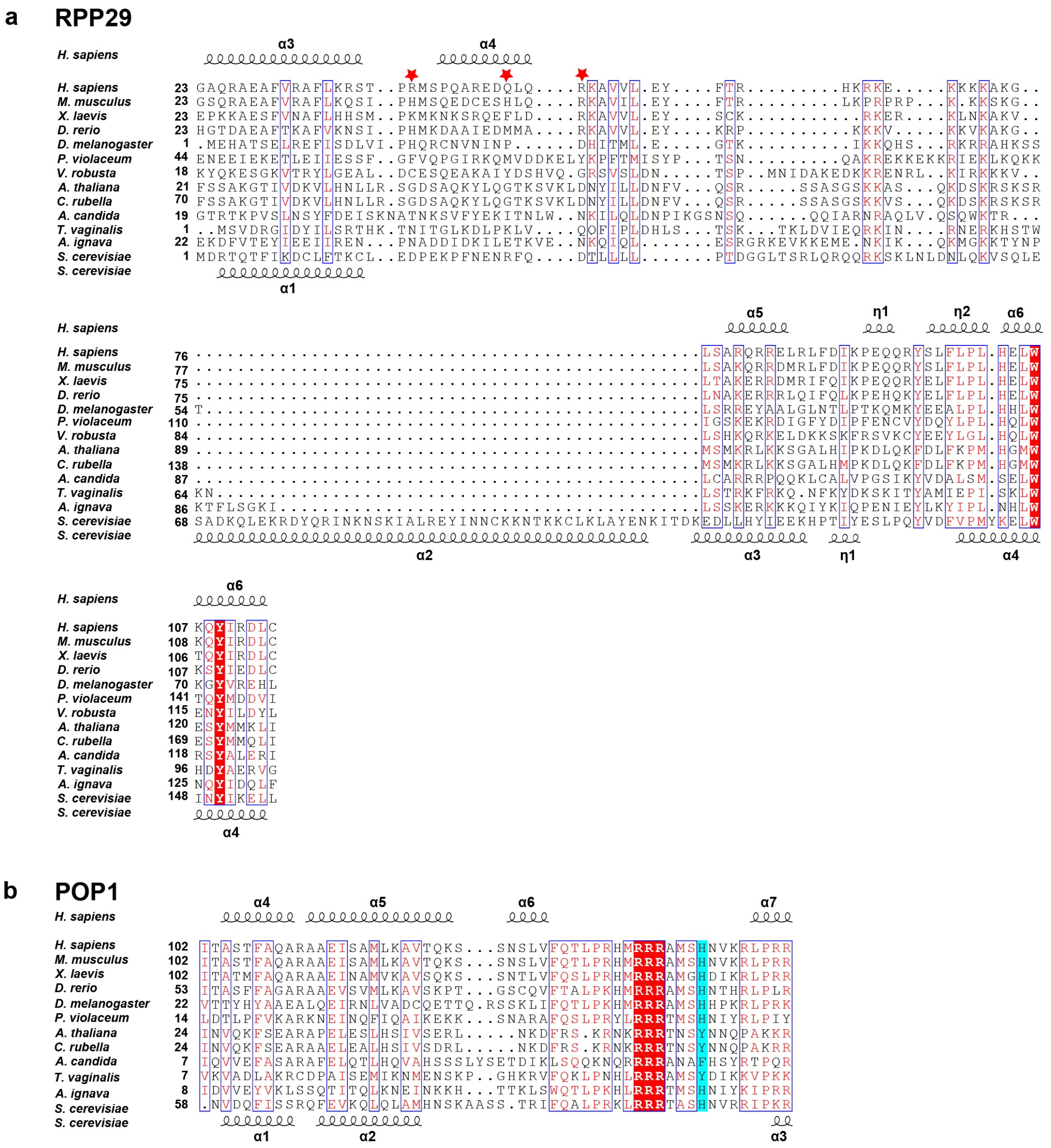

Supplementary Fig. 11 Multiple sequence alignment of RPP29$_{NTM}$ and POP1$_{NTM}$. a-b, Structural-based sequence alignment of RPP29$_{NTM}$ (**a**) and POP1$_{NTM}$ (**b**) from representative eukaryotic species. Conserved residues are colored in red. Residues in RPP29$_{NTM}$ of RNase MRP that help shape the ssRNA substrates in the catalytic groove are denoted with red asteroids. The key +4-anchor defining residue in POP1$_{NTM}$ is highlighted in cyan.

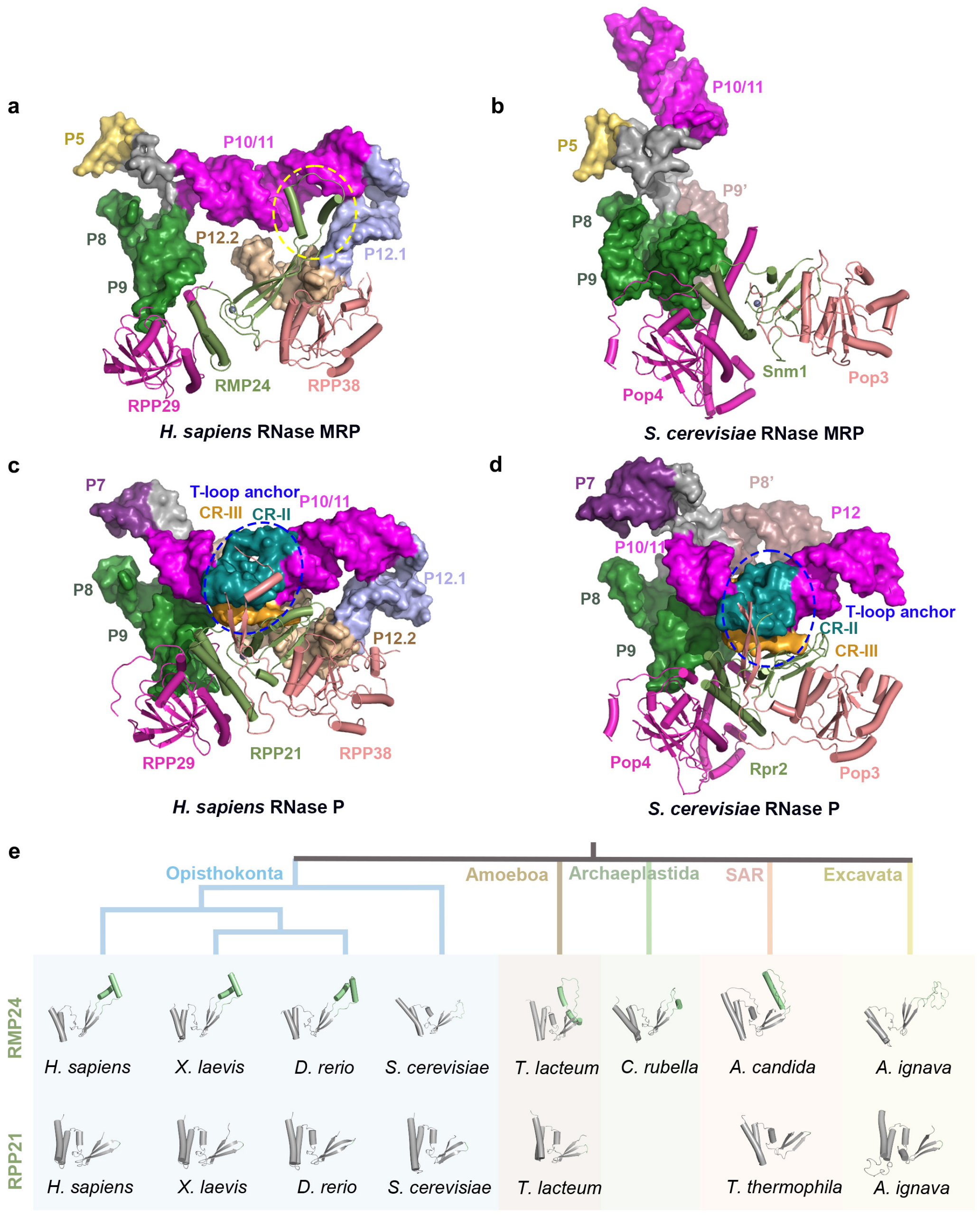

Supplementary Fig. 12 The RMP24-RPP29-RPP38 ternary module stabilizes the small lobe of RNase MRP. a-b, The RMP24-RPP29-RPP38 module stabilizes the small lobe of human (**a**) and yeast (**b**) RNase MRP. The RNA elements and the protein components are shown in surface and cartoon representations respectively, and colored as in Fig. 2. **c-d**, The RPP21-RPP29-RPP38 module forms a unique platform to hold

the CR-II/III T-loop anchor of human RPPH1 (**c**) and yeast Rpr1 (**d**) RNAs. The RNA elements and the protein components are shown in surface and cartoon representation respectively, and colored as in Fig. 2. The insertion in RMP24 is highlighted by a yellow dashed circle. **e**, Structural comparison of RPP21 and RMP24 across diverse eukaryotes. The phylogenetic tree (upper panel) illustrates the evolutionary relationship among the representative species. Representative RPP21 and RMP24 structures are predicted by AlphaFold-3 and shown in cartoon representation (lower panel). The insertion in the middle of RMP24 is highlighted in green.

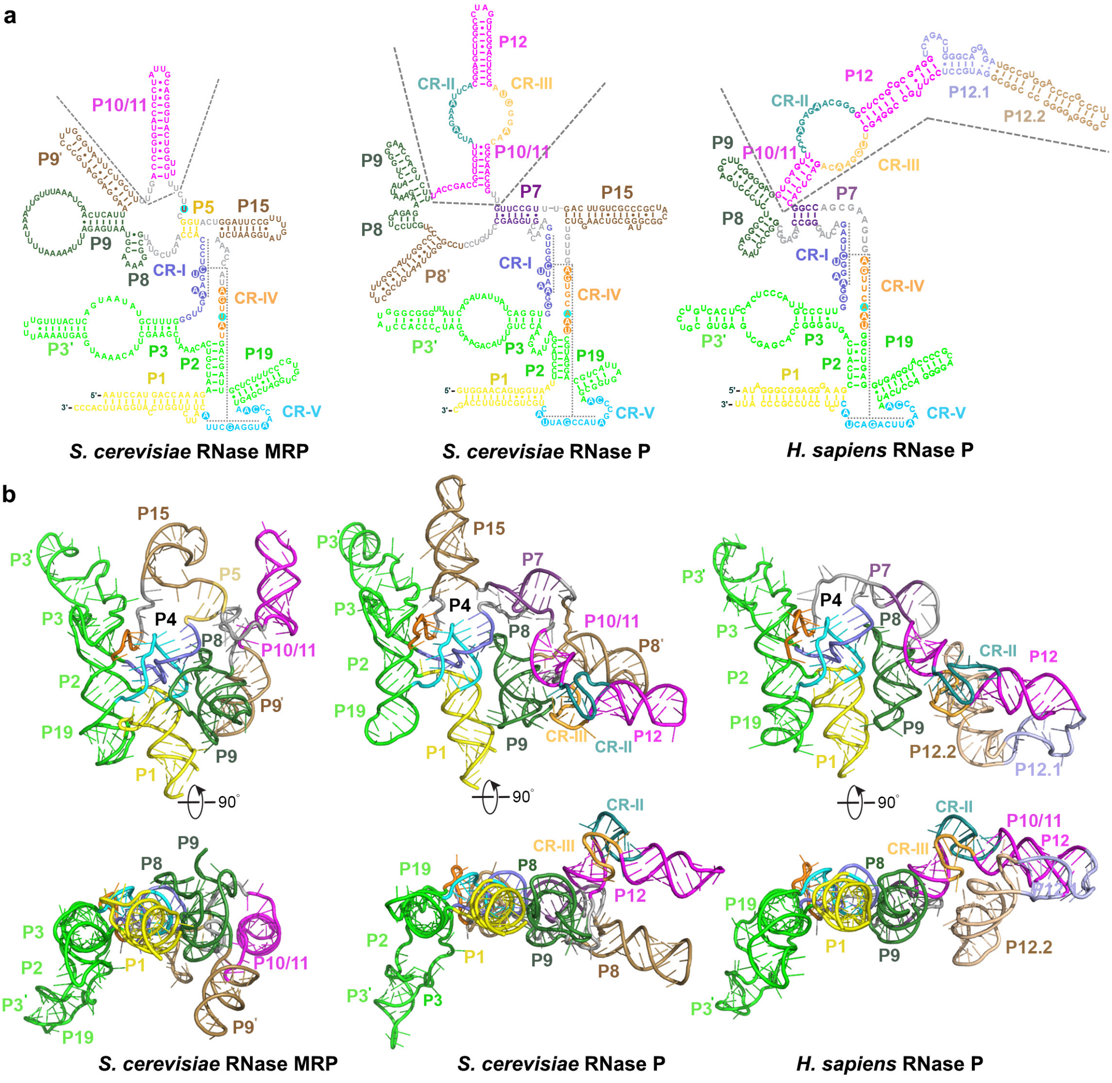

Supplementary Fig. 13 Structural comparison of the RNA components of human RNase P and yeast RNase P and MRP. a, 2D models of the RNA components of yeast RNase MRP (Nme1, First panel), yeast RNase P (Rpr1, Second panel), and human RNase P (RPPH1, Third panel). RNA stems are color coded (P1, yellow; P2, P3, P3', and P19, green; P5, yellow orange; P8 and P9, forest; P10/11, magenta; P12.1, light blue; P12.2, wheat). CR-I, CR-II, CR-III, CR-IV, and CR-V are colored in slate, deep tale, yellow orange, orange, and cyan, respectively. Nucleotides in CRs that are universally conserved are highlighted with shaded circles. Key −2 and +4 anchor

nucleotides in yeast RNase MRP ($U266^{Nme1}$ and $U226^{Nme1}$) and key −2 anchor nucleotides in yeast and human RNase P ($A314^{Rpr1}$ and $A274^{RPPH1}$) are highlighted with cyan circles. **b**, Two orthogonal views of the 3D structures of the RNA components corresponding to (a).

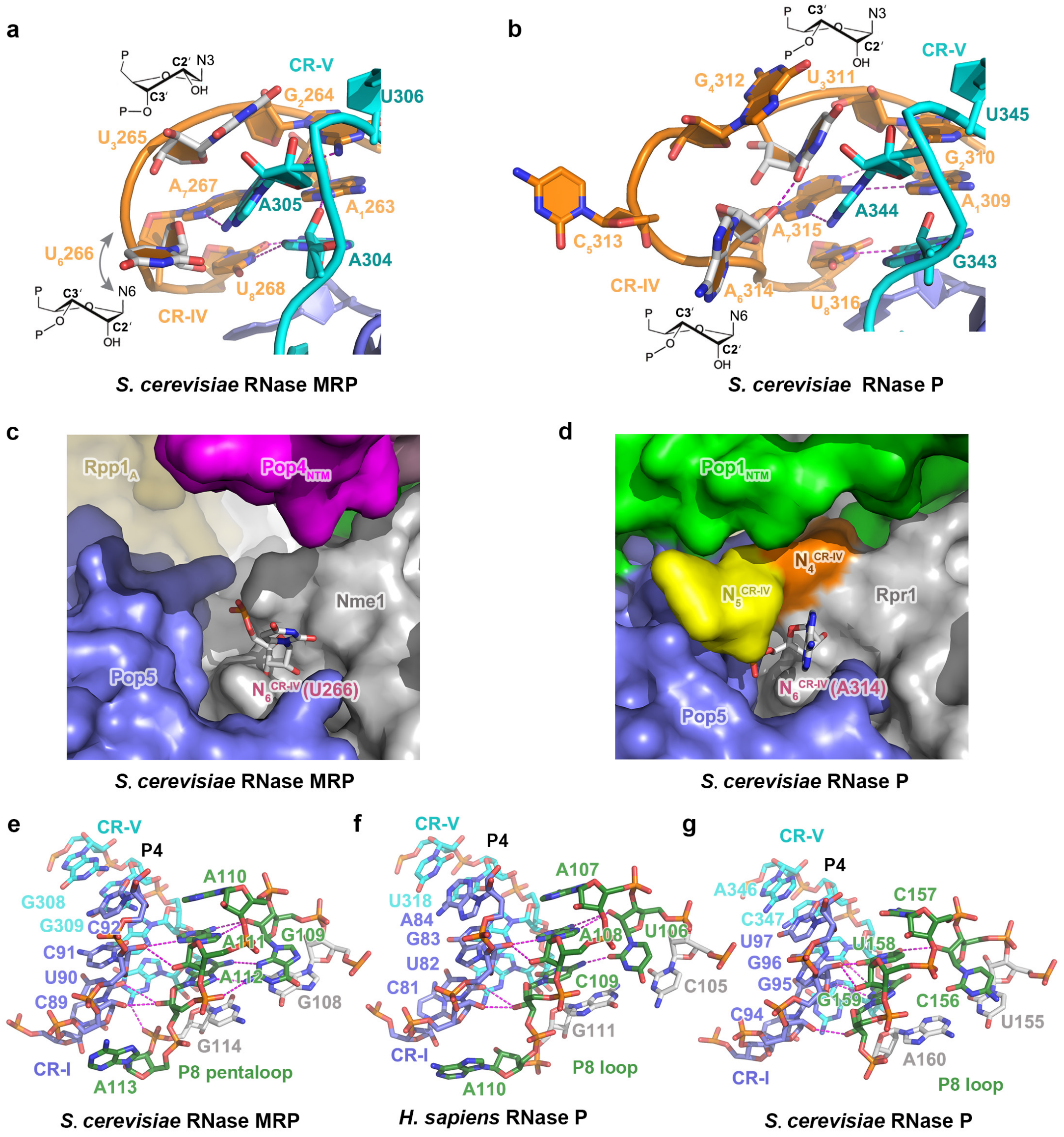

Supplementary Fig. 14 Unique structural features in yeast RNase MRP RNA. a-b, The U-shaped CR-IV of yeast Nme1 (**a**) and yeast Rpr1 (**b**) in cartoon representation. The sugar pucker of $N_3^{CR\text{-}IV}$ and $N_6^{CR\text{-}IV}$ are shown. Hydrogen-bonding interactions are denoted as magenta dashed lines. **c-d**, Comparison of yeast Nme1 $N_6^{CR\text{-}IV}$ (**c**) and Rpr1 $N_6^{CR\text{-}IV}$ (**d**). The $N_6^{CR\text{-}IV}$ nucleotides are shown in stick representation. The protein and the RNA components are shown in surface representation. **e-g**, Detailed structures of the contact between stem P4 and the stem P8 loop in yeast RNase MRP (**e**), human

RNase P (**f**), and yeast RNase P (**g**). CR-I, CR-V, and the P4 stem are labeled. Hydrogen bonds and electrostatic interactions are denoted as magenta dashed lines.

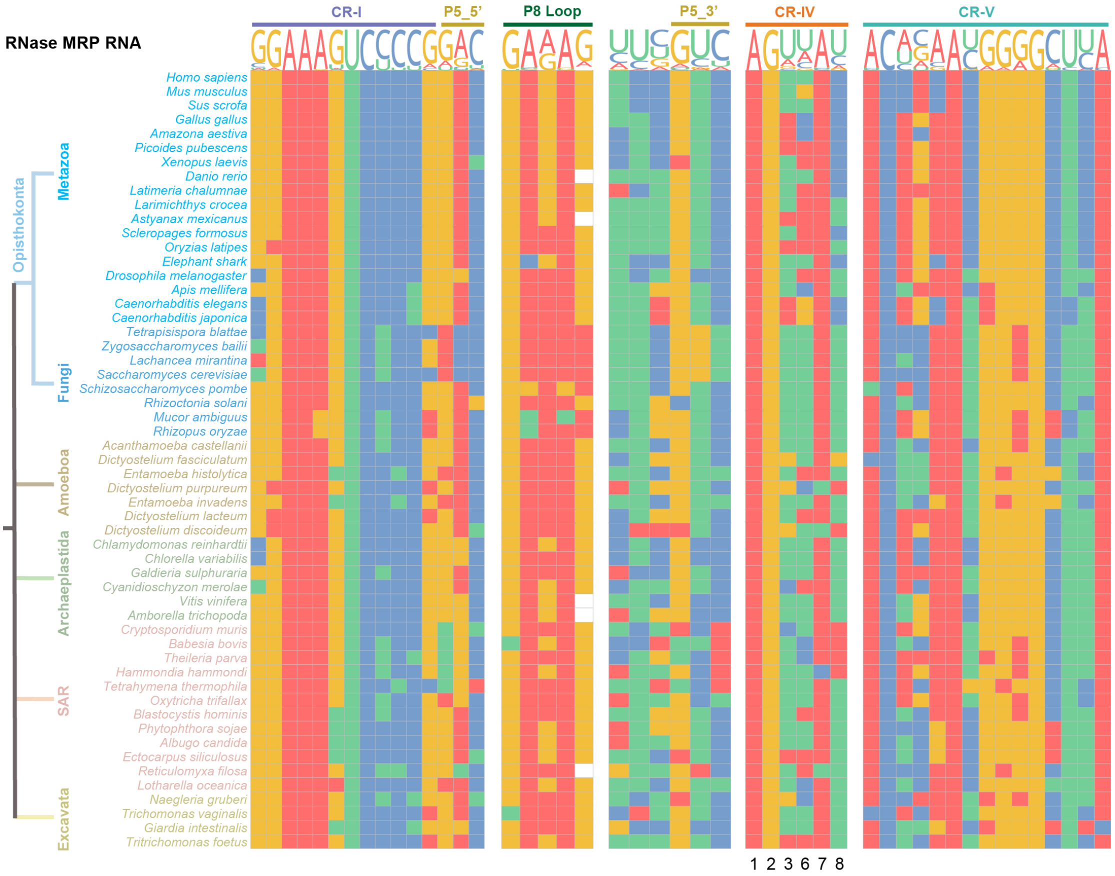

Supplementary Fig. 15 Sequence alignment of RNase MRP RNAs across eukaryotic species from Metazoan, Fungi, Amoebozoa, Archaeplastida, SAR, and Excavata. Nucleotide positions in CR-IV are labeled.

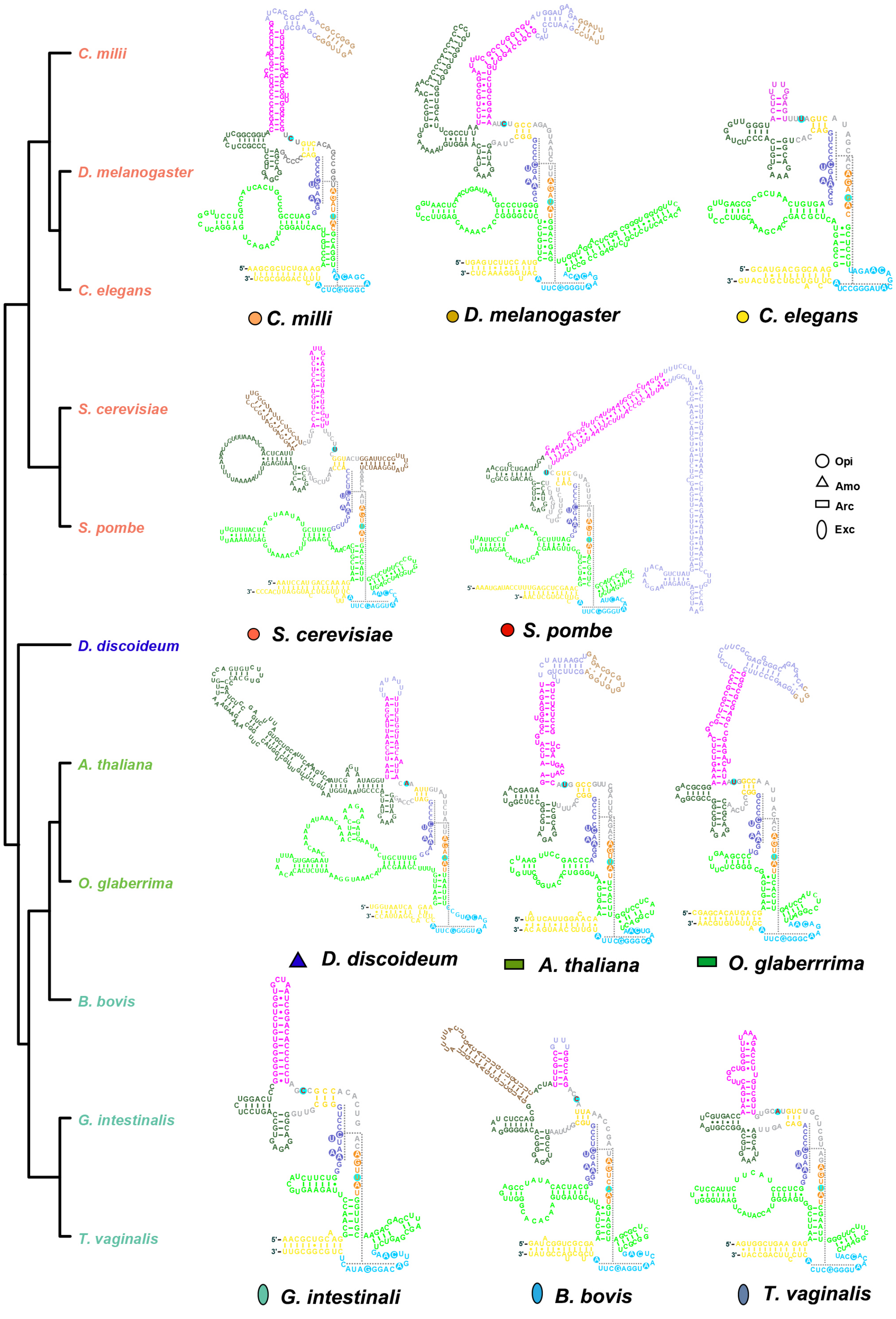

Supplementary Fig. 16 2D models of RNase MRP in representative eukaryotic species. The phylogenetic tree (left) illustrates the evolutionary relationships among

representative eukaryotic species. Key substrate-anchoring motifs are annotated: +4 anchor (red letters with cyan shading), -2 anchor (highlighted with cyan shading).

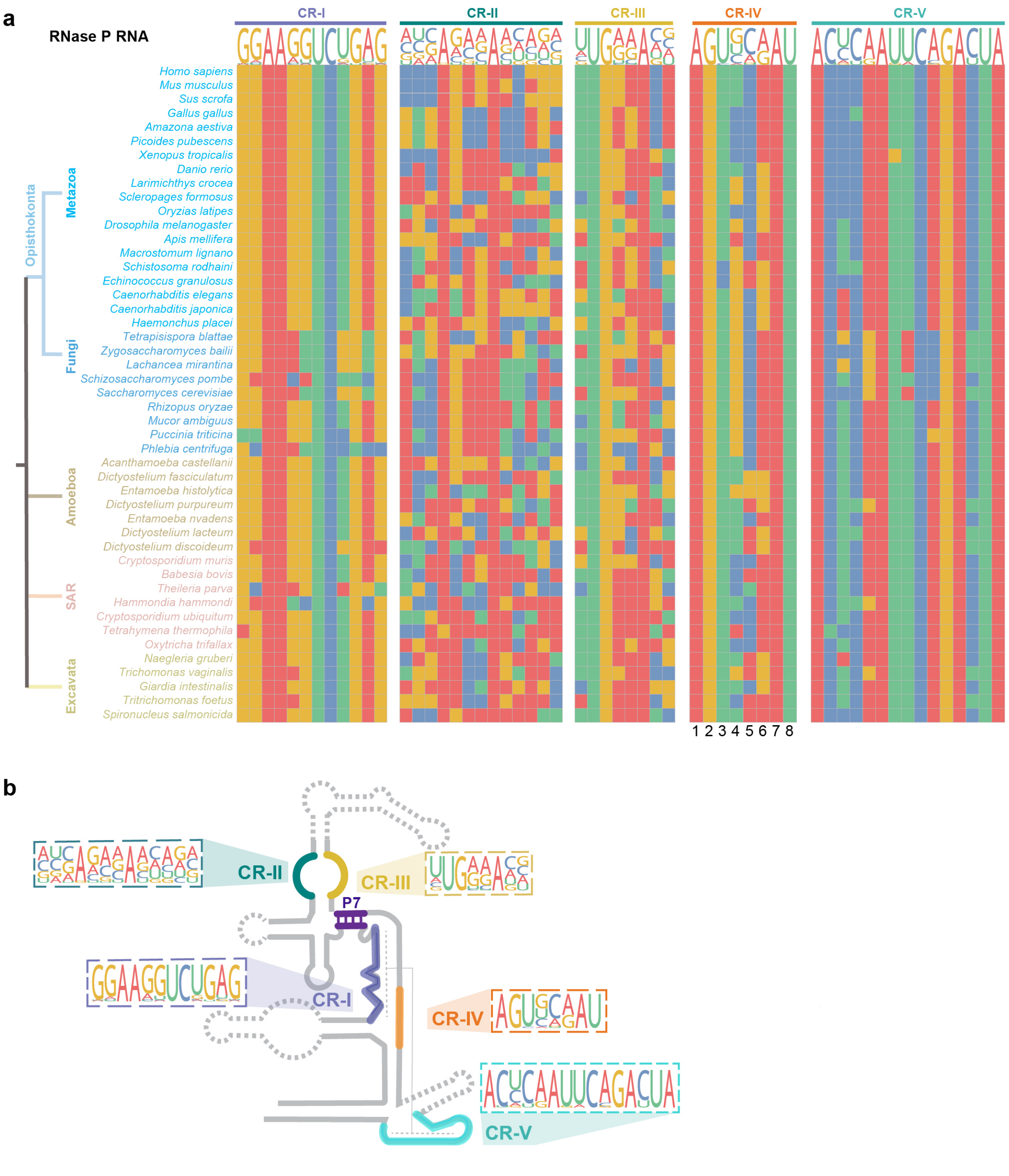

Supplementary Fig. 17 Evolutionary conservation of structural motifs in RNase P RNA. **a**, Sequence alignment of RNase P RNAs across eukaryotic species from Metazoan, Fungi, Amoebozoa, Archaeplastida, SAR, and Excavata. Nucleotide positions in CR-IV are labeled. **b**, Human RNase P RNA RPPH1 shown in a simplified secondary structure with highlighted conserved elements. Logo representations of CR-I, CR-II, CR-III, CR-IV, and CR-V across eukaryotic species from Metazoan, Fungi,

Amoebozoa, Archaeplastida, SAR, and Excavata.

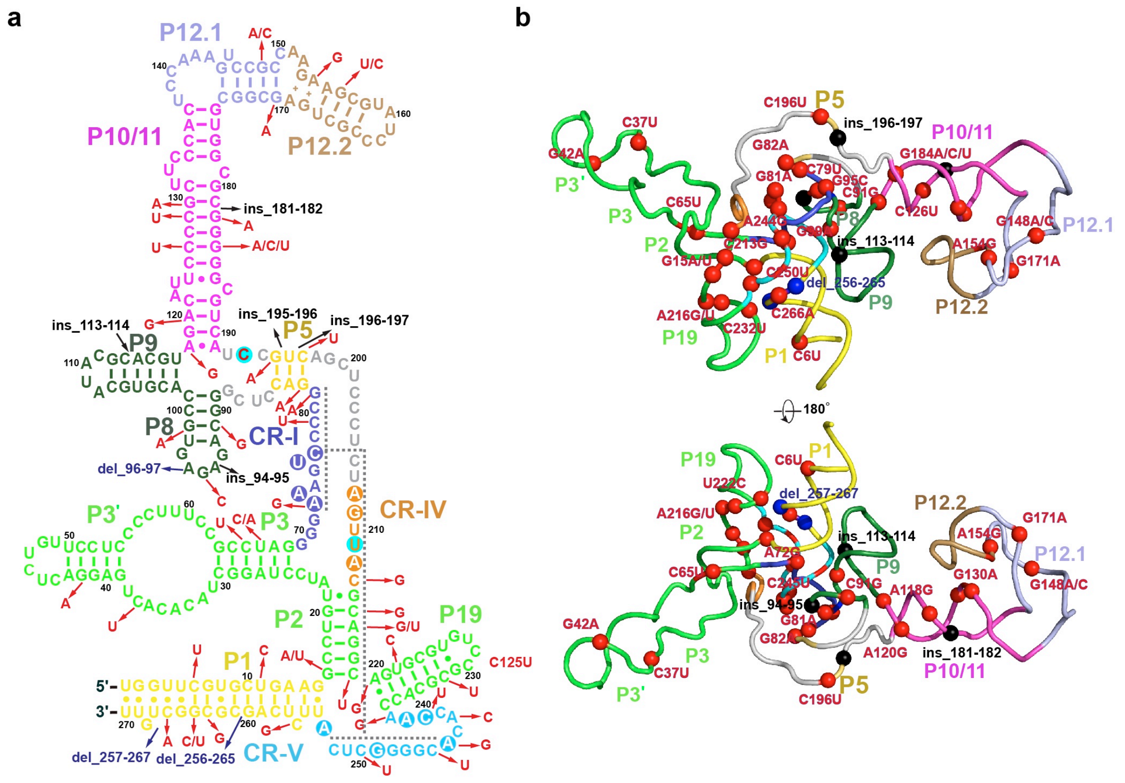

Supplementary Fig. 18 Disease-associated mutations in human RMRP. a, 2D model of RMRP labeled with mutations in human diseases. Single-nucleotide substitutions, insertion, and deletion mutations are denoted with red, black, and blue arrows, respectively. **b**, Human disease RMRP mutations are mapped onto the 3D structure of RMRP. Single-nucleotide substitutions are shown in the red sphere. The positions of insertion and deletion mutations are shown in black and blue spheres, respectively. Multiple nucleotide duplication mutations and mutations in the promoter region are not shown in this figure.

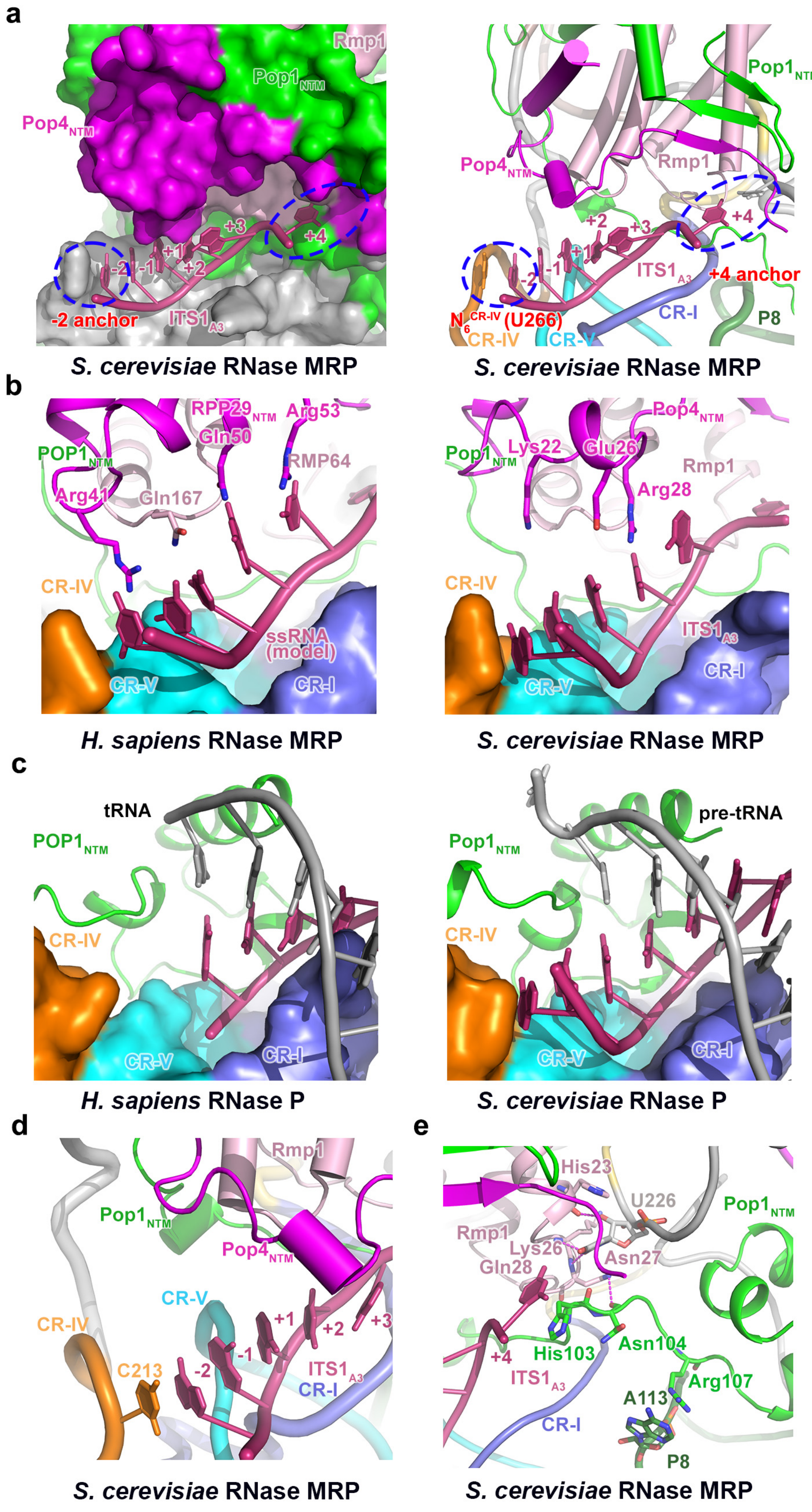

a
Rmp1
Pop1NTM
Pop4NTM
+3
+4
-2
-1
+1
+2
-2 anchor
ITS1A3
S. cerevisiae RNase MRP
Pop1NTM
Pop4NTM
Rmp1
+2
+3
+4
-2
-1
+1
ITS1A3
+4 anchor
N6CR-IV (U266)
CR-IV
CR-V
CR-I
P8
S. cerevisiae RNase MRP
b
RPP29NTM
Arg53
Gln50
POP1NTM
RMP64
Arg41
Gln167
CR-IV
ssRNA (model)
CR-V
CR-I
H. sapiens RNase MRP
Pop4NTM
Pop1NTM
Lys22
Glu26
Rmp1
Arg28
CR-IV
ITS1A3
CR-V
CR-I
S. cerevisiae RNase MRP
c
tRNA
POP1NTM
CR-IV
CR-V
CR-I
H. sapiens RNase P
pre-tRNA
Pop1NTM
CR-IV
CR-V
CR-I
S. cerevisiae RNase P
d
Rmp1
Pop1NTM
Pop4NTM
CR-V
CR-IV
C213
+1
+2
+3
-2
-1
ITS1A3
CR-I
S. cerevisiae RNase MRP
e
His23
U226
Pop1NTM
Rmp1
Lys26
Asn27
Gln28
+4
His103
Asn104
ITS1A3
Arg107
A113
CR-I
P8
S. cerevisiae RNase MRP

Supplementary Fig. 19 The Double-anchor substrate-binding mechanism of eukaryotic RNase MRP and the comparison with the substrate-binding mechanism of eukaryotic RNase P. a, Substrate-binding groove of yeast RNase MRP. The ssRNA substrate-binding groove is shown in the surface (left panel) and ribbon (right panel) representation. The $ITS1_{A3}$ substrate is shown in cartoons and colored in warm pink. The -2 and +4 anchors are denoted in blue dashed circles. **b**, $RPP29_{NTM}$ and RMP64 in human (left panel) and yeast (right panel) RNase MRP constrain the conformation of ssRNA substrates in the catalytic groove to mimic the to-be-processed strand of pre-tRNA acceptor stem in the RNase P active site. Critical residues on the inner surface of the substrate-binding groove that mediate substrate positioning are highlighted in stick representation. **c**, Left panel: conformation of the processed strand of pre-tRNA acceptor stem post cleavage in human RNase P active site. Right panel: conformation of the to-be-processed strand of pre-tRNA before cleavage in the yeast RNase P active site. **d**, Cartoon representation of -2 anchor (left panel), and +4 anchor (right panel) of yeast RNase MRP.

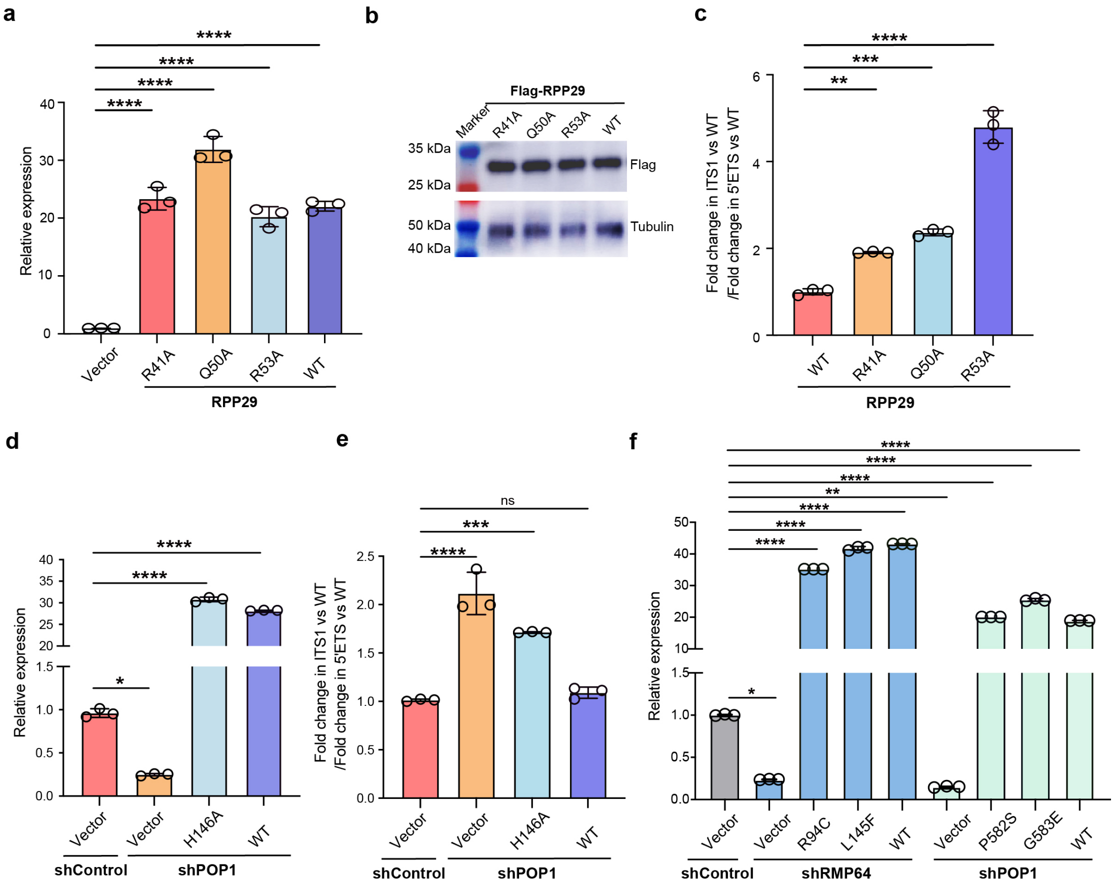

Supplementary Fig. 20 Functional analysis of human RNase MRP. a, Quantitative RT-PCR analysis of relative expression levels of overexpressed WT and mutant RPP29. **b**, Western blot analysis of Flag-tagged WT and mutant RPP29. **c**, Quantitative RT-PCR analysis revealed elevated pre-cleaved ITS1 levels relative to the 5'-external transcribed spacer (5' ETS) in RPP29 mutant-expressing cells compared to control cells. Both pre-cleaved ITS1 and the 5' ETS signals were normalized to 18S. **d**, Quantitative RT-PCR analysis of the expression levels of wild-type (WT) or the H146A mutant of POP1 in control cells, and in POP1-knockdown cells expressing empty vector, WT POP1, or the H146A mutant of POP1. **e**, Quantitative RT-PCR analysis of pre-cleaved ITS1 levels relative to the 5’-external transcribed spacer (5’-ETS) in control cells, and

in POP1-knockdown cells expressing empty vector, WT POP1, or the H146A mutant of POP1. **f**, Quantitative RT-PCR analysis of the expression levels of WT or disease-causing mutants of RMP64 and POP1 in control cells, in RMP64-knockdown cells expressing empty vector, WT RMP64, or the R94C and L145F mutants of RMP64, and in POP1-knockdown cells expressing empty vector, WT POP1, or the P582S and G583E mutants of POP1. For all experiments, data were shown as mean ± SD from three independent replicates. Significance was determined using One-way ANOVA. *$p < 0.05$, **$p < 0.01$, ***$p < 0.001$, ****$p < 0.0001$.

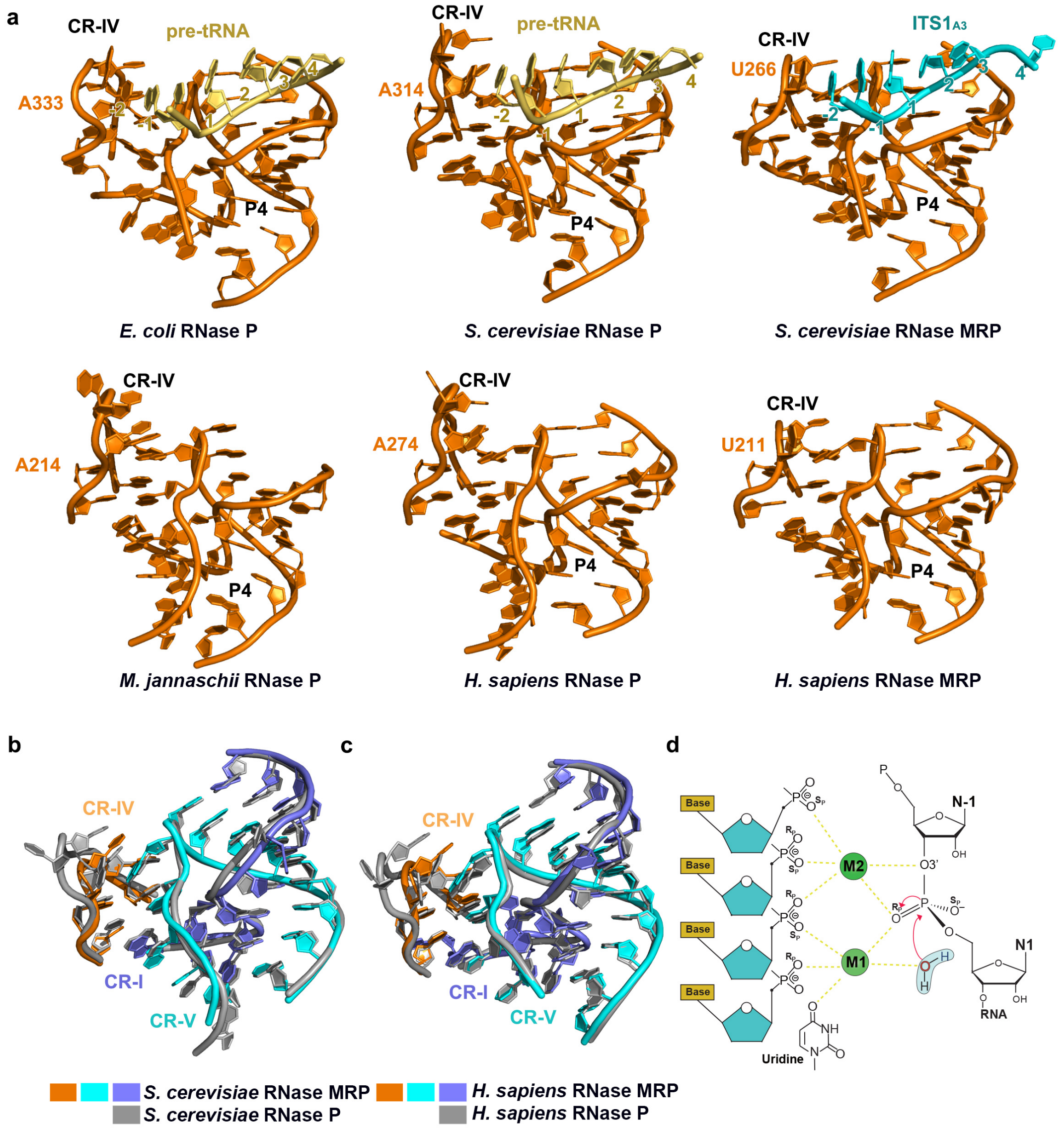

Supplementary Fig. 21 Conserved S_N2 catalytic mechanism of RNase P and MRP.

a, Active site architecture comparison of bacterial, archaeal, and eukaryotic RNase P and eukaryotic RNase MRP. Substrates in the active sites of RNase P and MRP are colored in yellow and cyan, respectively. The figures were based on determined high-resolution structures with PDB codes 7UO1 (*Escherichia coli* RNase P), 6K0A (*Methanococcus jannaschii* RNase P), 6AH3 (*Saccharomyces cerevisiae* RNase P), 7C7A (*Saccharomyces cerevisiae* RNase MRP), 6AHR (*Homo sapiens* RNase P), and

9UH7 (*Homo sapiens* RNase MRP). **b-c**, Catalytic center comparison of RNase P and MRP complexes. Structural superposition of yeast (**b**) and human (**c**) RNase P and MRP catalytic centers. CR-I, CR-IV, and CR-V in RNase P are colored gray, and those in RNase MRP are in slate, orange, and cyan, respectively. **d**, Conserved two-metal-ion catalytic mechanism. Proposed S_N2-type catalytic mechanism of RNase P and MRP. The reactive oxygen is colored in magenta, the substrate scissile phosphate is depicted in a transition state, and the interactions mediated by the catalytically important Mg^{2+} ions (M1 and M2) are shown as yellow-green dashed lines.

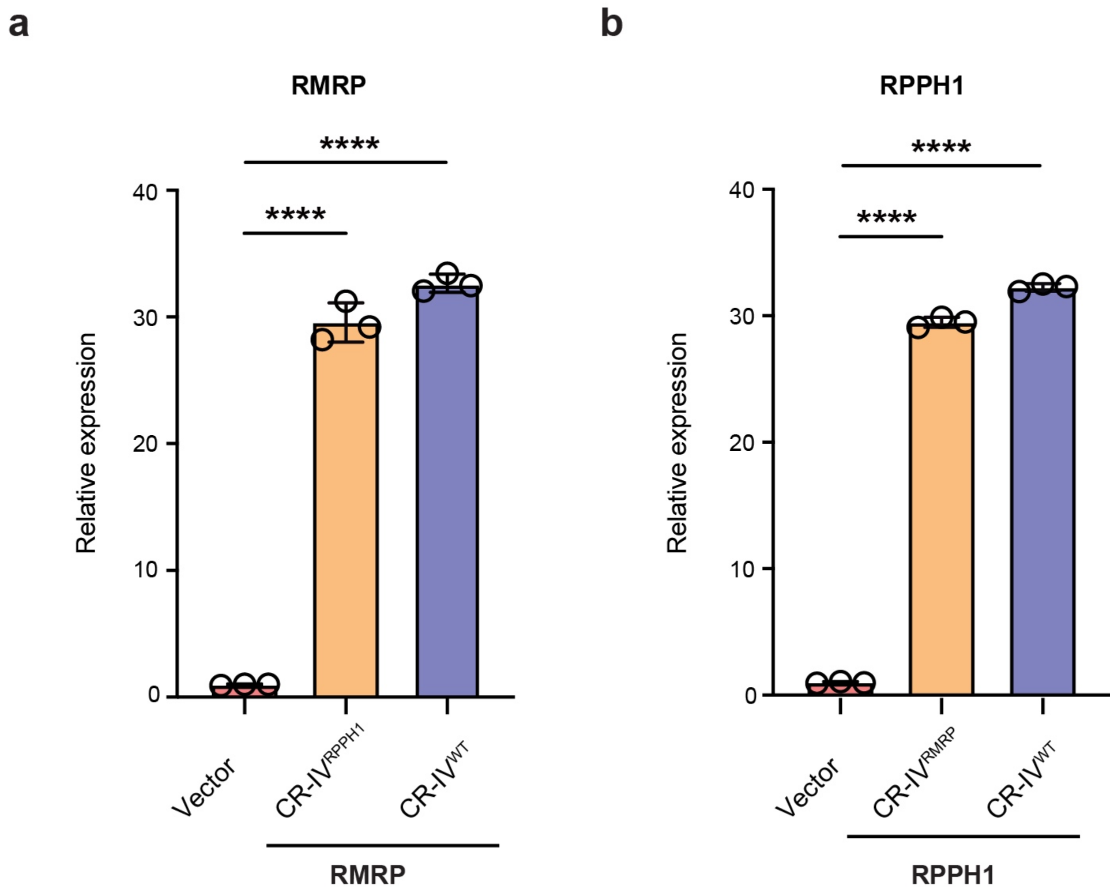

Supplementary Fig. 22 Functional analysis of CR-IV swapping between human RNase P and MRP. a, Relative expression levels of overexpressed RMRP RNAs: CR-IV^{RPPH1} (RMRP CR-IV replaced by RPPH1 CR-IV) versus CR-IV^{WT} (wild-type RMRP CR-IV). **b**, Relative expression levels of overexpressed RPPH1 RNAs: CR-IV^{RMRP} (RPPH1 CR-IV replaced by RMRP CR-IV) versus CR-IV^{WT} (wild-type RPPH1 CR-IV). For all experiments, data were shown as mean ± SD from three independent replicates. Significance was determined with a two-tailed Student's t-test. ****$p < 0.0001$.

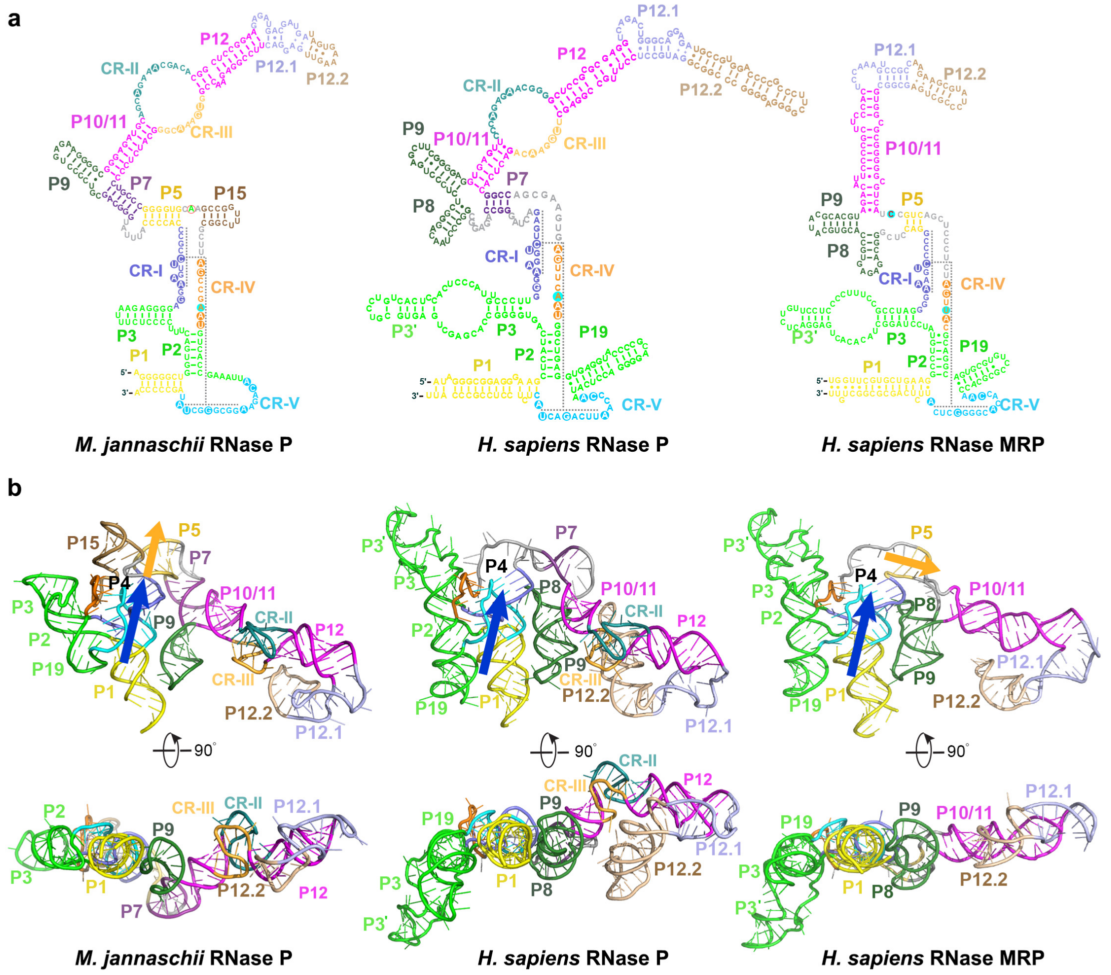

Supplementary Fig. 23 Evolution of the catalytic RNA components of eukaryotic RNase P and MRP. a, 2D models of the RNA components in archaeal *Methanocaldococcus jannaschii* RNase P (left panel), human RNase P (middle panel), and human RNase MRP (right panel). Conserved regions CR-I, CR-IV, and CR-V are highlighted. **b**, Two orthogonal views of 3D structures of the RNA components corresponding to (a). Directions of stem P4 and P5 are denoted by blue and orange arrows, respectively.

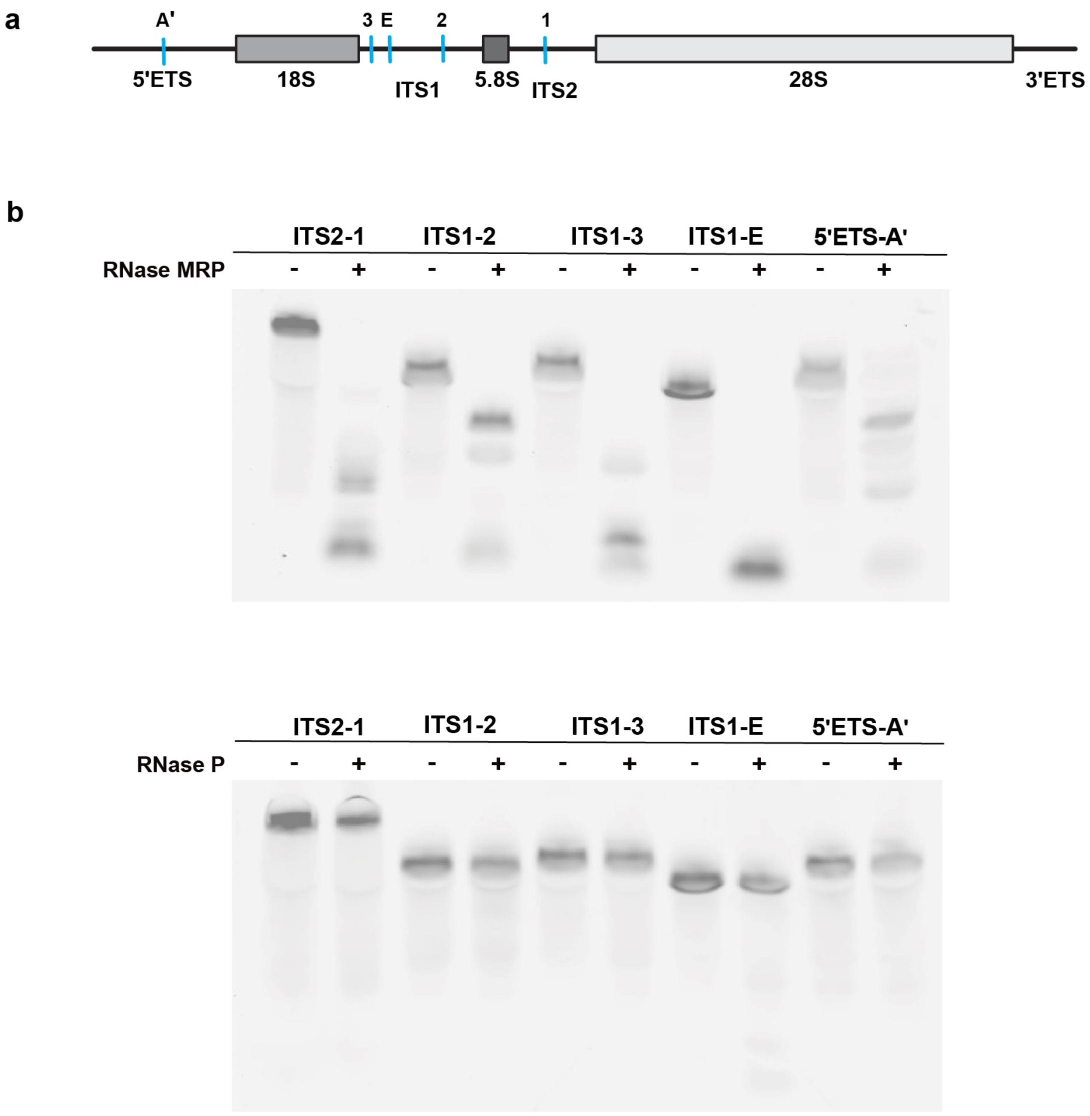

Supplementary Fig. 24 *In vitro* cleavage assay of purified human RNase MRP holoenzyme. a, Schematic diagram of previously reported cleavage sites within pre-rRNA by human RNase MRP[4]. The predicted cleavage sites ITS1-A', ITS1-3, ITS1-E, ITS1-2, and ITS2-1 are denoted. **b**, *In vitro* cleavage activity analysis of various pre-rRNA substrates designed based on the sites shown in panel (a) by purified human RNase MRP (upper panel) and human RNase P (lower panel). Detailed substrate sequences are listed in the Methods section.

Supplementary Table 1. Cryo-EM data collection, refinement, and validation statistics.

	RNase MRP (Large_lobe) EMD-64157 PDB 9UH7	RNase MRP (Holoenzyme) EMD-64159 PDB 9UH9	RNase MRP (Small_lobe) EMD-64160 PDB 9UHA
**Data collection and processing**			
Magnification	81,000	81,000	81,000
Voltage (kV)	300	300	300
Electron exposure (e–/Å^2)	50	50	50
Defocus range (μm)	1.5-2.2	1.5-2.2	1.5-2.2
Pixel size (Å)	1.1	1.1	1.1
Symmetry imposed	C1	C1	C1
Initial particle images (no.)	3,828,791	3,828,791	3,828,791
Final particle images (no.)	777,423	432,377	294,518
Map resolution (Å)	2.84	3.47	3.93
FSC threshold	0.143	0.143	0.143
Map resolution range (Å)	2.5-4.5	3.0-6.5	3.5-6.5
**Refinement**			
Initial model used (PDB code)	n/a	n/a	n/a
Model resolution (Å)	2.8	3.5	3.9
FSC threshold	0.143	0.143	0.143
Map sharpening *B* factor (Å^2)	-100	-20	-30
Model composition			
Non-hydrogen atoms	24,267	27,111	27,442
Protein residues	2,519	2,741	2,741
Nucleotides	204	254	270
Ions	-	1	1
B factors (Å^2)			
Protein	34.1	39.9	97.6
Nucleotide	45.1	60.0	93.4
Ion	-	106.2	147.5
R.m.s. deviations			
Bond lengths (Å)	0.003	0.002	0.002
Bond angles (°)	0.670	0.621	0.703
Validation			
MolProbity score	1.92	1.90	1.96
Clashscore	8.06	8.99	9.59
Poor rotamers (%)	1.91	1.58	1.66
Ramachandran plot			
Favored (%)	96.05	96.07	95.89
Allowed (%)	3.95	3.93	4.11
Disallowed (%)	0.00	0.00	0.00
