# Supplementary material for: Structural and evolutionary insights into the eukaryotic RNase MRP ribonucleoprotein complex
Source: Nat Commun. 2026 Mar 26;17:4451. doi: 10.1038/s41467-026-71007-9 (PMC13184271; doi:10.1038/s41467-026-71007-9)
Supplement: Supplementary file 2 — Description of Additional Supplementary Files [file 41467_2026_71007_MOESM2_ESM.pdf]

Description of Additional Supplementary Files

File name: Supplementary Data 1
Description: DALI search results using yeast Rmp1 core structure as a query to search the AlphaFold database of *Homo sapiens*, *Mus musculus*, *Danio rerio*, *Drosophila melanogaster*, *Caenorhabditis elegans* and *Arabidopsis thaliana*.

File name: Supplementary Data 2
Description: DALI search results using yeast Snm1 core structure as a query to search the AlphaFold database of *Homo sapiens*, *Mus musculus*, *Danio rerio*, *Drosophila melanogaster*, *Dictyostelium discoideum* and *Arabidopsis thaliana*.

File name: Supplementary Data 3
Description: Foldseek search results using yeast Rmp1 and Snm1 and human NEPRO (RMP64) and C18orf21 (RMP24) as queries.

File name: Supplementary Data 4
Description: IP-MASS data information.

File name: Supplementary Data 5
Description: Real-time quantitative PCR primers and shRNA used in this study.
